# DNA Methylation of the Gonadal Aromatase (*cyp19a*) Promoter Is Involved in Temperature-Dependent Sex Ratio Shifts in the European Sea Bass

**DOI:** 10.1371/journal.pgen.1002447

**Published:** 2011-12-29

**Authors:** Laia Navarro-Martín, Jordi Viñas, Laia Ribas, Noelia Díaz, Arantxa Gutiérrez, Luciano Di Croce, Francesc Piferrer

**Affiliations:** 1 Institut de Ciències del Mar, Consejo Superior de Investigaciones Científicas (CSIC), Barcelona, Spain; 2 Centre de Regulació Genòmica (CRG)/ICREA and Univeristat Pompeu Fabra (UPF), Barcelona, Spain; Queensland Institute of Medical Research, Australia

## Abstract

Sex ratio shifts in response to temperature are common in fish and reptiles. However, the mechanism linking temperature during early development and sex ratios has remained elusive. We show in the European sea bass (sb), a fish in which temperature effects on sex ratios are maximal before the gonads form, that juvenile males have double the DNA methylation levels of females in the promoter of gonadal aromatase (*cyp19a*), the enzyme that converts androgens into estrogens. Exposure to high temperature increased the *cyp19a* promoter methylation levels of females, indicating that induced-masculinization involves DNA methylation-mediated control of aromatase gene expression, with an observed inverse relationship between methylation levels and expression. Although different CpGs within the sb *cyp19a* promoter exhibited different sensitivity to temperature, we show that the increased methylation of the sb *cyp19a* promoter, which occurs in the gonads but not in the brain, is not a generalized effect of temperature. Importantly, these effects were also observed in sexually undifferentiated fish and were not altered by estrogen treatment. Thus, methylation of the sb *cyp19a* promoter is the cause of the lower expression of *cyp19a* in temperature-masculinized fish. *In vitro*, induced methylation of the sb *cyp19a* promoter suppressed the ability of SF-1 and Foxl2 to stimulate transcription. Finally, a CpG differentially methylated by temperature and adjacent to a Sox transcription factor binding site is conserved across species. Thus, DNA methylation of the aromatase promoter may be an essential component of the long-sought-after mechanism connecting environmental temperature and sex ratios in vertebrate species with temperature-dependent sex determination.

## Author Summary

Temperature changes during early embryonic and/or larval stages are able to modify sex ratios in fish and reptiles. However, the underlying mechanism by which temperature is able to modify the molecular pathways that developing gonads follow to become ovaries or testes is still unknown. One of the most interesting questions raised from previous studies with our model species, the European sea bass, was how temperature could affect the developmental fate of the gonads at a time when they were not even formed in the most rudimentary manner. This was the telltale sign of an epigenetic mechanism. In this study, DNA methylation levels of the aromatase promoter were analyzed in European sea bass exposed to different temperatures during early developmental stages. Aromatase is the enzyme that converts androgens (male hormones) into estrogens (female hormones), which are essential for ovarian development in all non-mammalian vertebrates. We show that increased temperature during a critical period in early development is able to increase DNA methylation of the aromatase promoter, preventing aromatase gene expression. We conclude that gonadal aromatase promoter methylation is most likely part of the long-sought-after mechanism connecting temperature and environmental sex determination in vertebrates.

## Introduction

The sex ratio is a crucial demographic parameter important for population viability that is established by the processes of sex determination and differentiation. The sex determination mechanisms in vertebrates include genotypic sex determination (GSD), temperature-dependent sex determination (TSD) or a combination of both. In TSD, the temperature experienced during a particular time during early development, referred to as the thermosensitive period (TSP), irreversibly determines gonadal sex. TSD is well established in reptiles and fish [1]. Regardless of the sex determining system, in non-mammalian vertebrates the androgen-to-estrogen ratio determines whether a sexually undifferentiated gonad sexually differentiates into a testis or ovary. This sex steroid ratio depends of the activity of the enzyme aromatase, Cyp19a, the product of the *cyp19a* gene, which irreversibly converts androgens into estrogens. Further, in ectothermic vertebrates, the effects of environmental temperature on sex ratios are mediated by changes in *cyp19a* expression. Thus, in reptiles with TSD, exposure to female-promoting temperatures is invariably associated with gonadal *cyp19a* upregulation, whereas exposure to male-producing temperatures is associated with *cyp19a* suppression [2], [3]. In all fish species analyzed so far, more males are produced with increasing temperatures [4]. The masculinizing effects of high temperature are also invariably caused by an inhibition of *cyp19a* expression and enzymatic activity [5]–[7]. Thus, regardless of the animal group and the sex determining mechanism considered, *cyp19a* regulation is a key player in the sex ratio response to temperature in vertebrates. Unfortunately, the molecular mechanism by which temperature affects *cyp19a* has remained elusive [1], [8], and this is most important since identifying environmental cues and their perception and transduction mechanisms is a central focus of eco-devo research [9].

Gorelick [10] hypothesized that different methylation patterns of virtually identical sex chromosomes in species with TSD could be altered by small environmental changes, hence determining the sex of individuals. He also proposed that sex differences are initially determined by different patterns of methylation on nuclear DNA of females and males. Recently, reviewing the evidence gathered so far on DNA methylation of four steroidogenic enzymes, it has been postulated [11] that epigenetics are the missing link between genetics, the environment and endocrine functions. Furthermore, other recent studies [12] have shown epigenetic regulation not only of the enzymes involved in the steroidogenic pathway but also of some transcription factors and nuclear receptors related to steroid biosynthesis and action. In mammals, *Cyp19* is expressed in a tissue-specific manner and regulated by different tissue-specific promoters [13]. Recently, epigenetic regulation of *Cyp19* gene expression in mammals has been demonstrated in humans [14], cattle and sheep [15], [16], and buffalo [17].

The European sea bass (sb), *Dicentrarchus labrax*, is a gonochoristic GSD+TE species, which means that sex determination can be controlled by both genetic (GSD) and temperature effects (TE). Temperature and genetics contribute approximately equally to sex determination [18]–[20]. Importantly, both GSD+TE and “pure” TSD fish species have exactly the same response to high temperature: inhibition of *cyp19a* expression [4]. Thus, the sea bass is a perfectly suited model and indeed one of the best documented fish species in terms of sex ratio shifts in response to temperature [20]–[25]. Exposure to high temperatures (>17°C) during the TSP, which covers the period between fertilization to ∼60 days post fertilization (dpf), results in male-biased sex ratios (Figure S1) [20]. During sex differentiation, the primordial gonads, starting from a common primordium, can take two mutually exclusive different developmental pathways towards the formation of an ovary or a testis. Previous studies demonstrated that temperature effects in the European sea bass are more pronounced during the first half of the TSP, when fish are about 30 mm [20]. Interestingly, this not only occurs before morphological sex differentiation takes place (>150 dpf; ∼120 mm fish) but also even before the formation of the gonadal ridges at ∼35 dpf [26]. This demonstrates that the time when the temperature influence takes place is well before the actual differentiation period of the gonads into either the male or the female pathway. For that reason, we hypothesized the existence of an epigenetic mechanism activated by temperature, which could result in different levels of DNA methylation in the gonadal *cyp19a* promoter, which in turn would affect gene expression, estrogen synthesis and hence sex ratios.

## Results

### Methylation levels of the European sea bass gonadal *cyp19a* promoter are sex-specific and influenced by the temperatures experienced during early life

**Figure 1:**
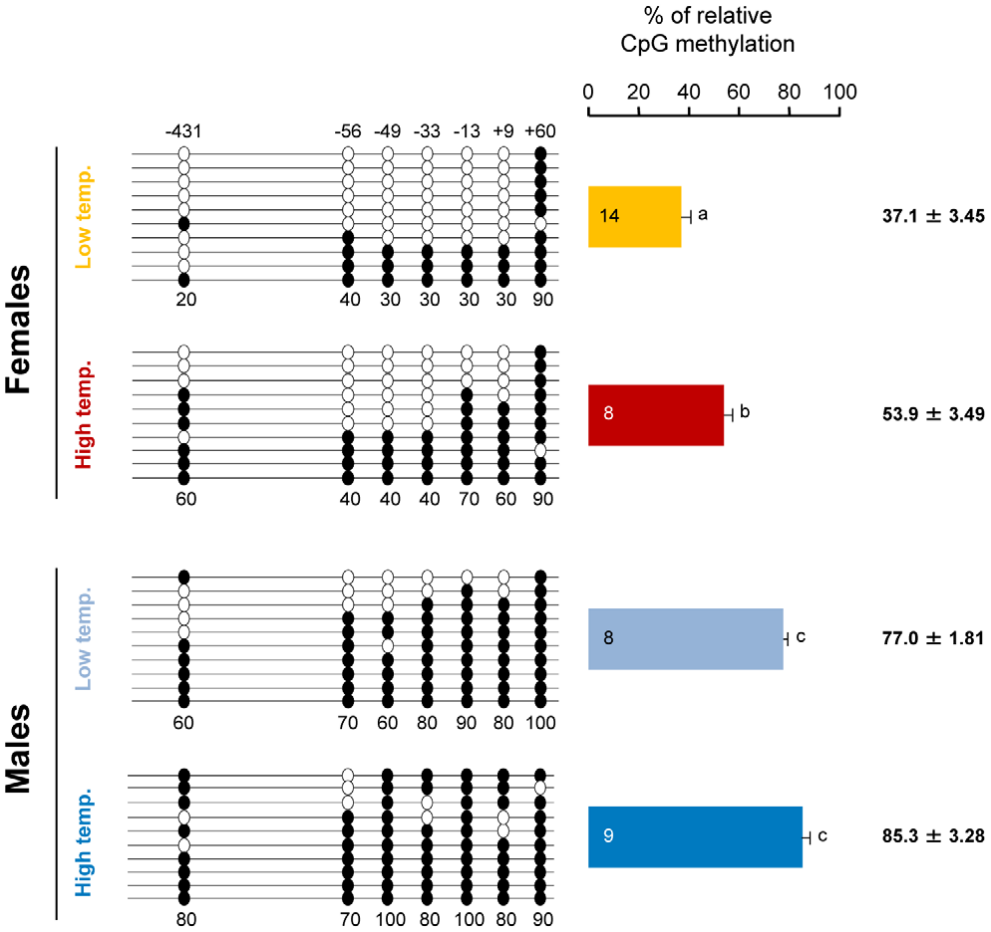
Resulting differences in sb *cyp19a* promoter methylation according to sex and temperature treatment. Typical methylation patterns of sea bass gonadal aromatase promoter of one-year-old sea bass females and males that were reared at low and high temperature during early development, as observed in this study. One fish for each sex and temperature combination representative of the level of methylation is shown. Numbers with a plus or minus sign indicate CpG positions with respect to the transcription starting site. Open and filled circles denote unmethylated or methylated positions, respectively. Ten clones per fish were analyzed. Average methylation was calculated specifically for each CpG position (number below each column). The number inside the bar indicates sample size. Results as mean ± SEM. Groups with different letters are significantly different (*P*<0.01).

To test our hypothesis, the previously characterized sea bass aromatase (sb *cyp19a*) promoter [27] was examined and the CpG dinucleotides ∼500 bp upstream of the transcription start site were selected (see Materials and Methods and Figure S2). First, gonadal methylation levels of the sb *cyp19a* promoter were determined using bisulfite sequencing in one-year-old sea bass males and females (family 1) exposed to two different temperature regimes during the first 60 days of life (high temperature, HT group, and low temperature, LT group; see Materials and Methods and Figure S3). A two-way ANOVA indicated significant differences in average DNA methylation levels of the sb *cyp19a* promoter in one year old sea bass (∼160 mm length; ∼73 g weight) according to sex (F = 118.2; *P* = 0.001) and temperature treatment (F = 14.6; *P* = 0.000), but without interaction between the two variables (*P* = 0.703). Results showed that, overall, males had twice as much sb *cyp19a* promoter DNA methylation levels as females (mean ± S.E.M.: 81.15±2.54% vs. 45.5±3.47%; two-tailed Student's *t*-test; t = −9.591, *P* = 0.000; Figure S4). Furthermore, sex-related differences were also clearly evident by distinct frequency distributions, with values in the range 12.9–72.8% for females and 71.4–97.1% for males, with a threshold value of 67% (Figure S4). The most important finding, however, was that exposure to high temperature increased gonadal sb *cyp19a* methylation levels in females from 37.1±3.45 to 53.9±3.49% (two-tailed *t*-test; t = 3.186, *P* = 0.005), and from 77.0±1.81 to 85.3±3.28% in males (two-tailed *t*-test, t = 2.056, *P* = 0.062; Figure 1).

**Figure 2:**
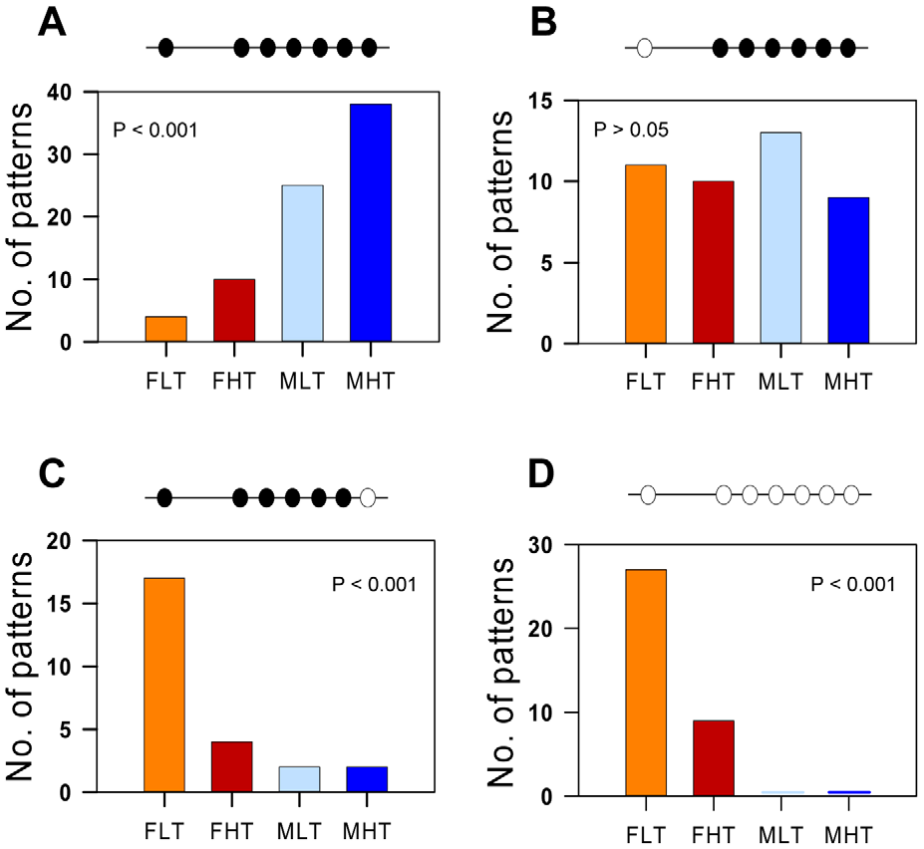
Absolute frequency of the four most frequently observed methylation patterns of the sb *cyp19a* promoter by sex and temperature treatment. In all cases the expected frequency of the patterns represented here per group was >5. A, Frequency of the methylation pattern consisting of all seven positions methylated. B, Frequency of the methylation pattern with the 5′first position (−431) unmethylated and the remaining six methylated. C, Frequency of the methylation pattern with the last position (+60) unmethylated and the remaining six methylated. D, Frequency of the methylation pattern with all positions unmethylated. Abbreviations: Females at Low Temperature (FLT); Females at High Temperature (FHT); Males at Low Temperature (MLT); Males at high temperature (MHT). The level of significance is indicated for each case.

With seven CpGs analyzed in the sb *cyp19a* promoter, the maximum number of possible methylation patterns was 128, i.e., 2^7^. Of these, 58 were observed, distributed with different absolute frequencies according to treatment (Figure S5). Based on the observed frequencies of each treatment, contingency analysis was applied to determine if there was any influence of sex and temperature in the distribution of methylation patterns. This, as statistical practice dictates, could be only done for those patterns in which the expected frequency for a given treatment was 5 or higher, which, with four groups means whose observed frequency was 20 or higher. This condition was satisfied by four patterns (Figure 2). These methylation patterns include one in which all seven positions were methylated, another in which all seven positions were unmethylated and two in which either the first or the last position was unmethylated and the remaining were methylated. Analysis of the presence of these four patterns among the four treatment groups showed highly significant differences (*P*<0.001) in three of them (Figure 2). This reinforces the observation that LT females had the lowest *cyp19a* promoter methylation levels since they have the highest frequency of the pattern with all positions unmethylated and, conversely, the patterns consisting of all positions methylated was most frequently present in the HT males (Figure 2). Technical error due to PCR bias was avoided since the number of mean methylation patterns was maintained through the different treatments in the range 5.6–7.1 out of a theoretically maximum range of 1–10. This indicates that the PCR reaction was able to amplify different methylation patterns or epialleles. (Figure S6A). Further still, the number of different methylation patterns was independent of the average *cyp19a* methylation levels (Figure S6B).

**Figure 3:**
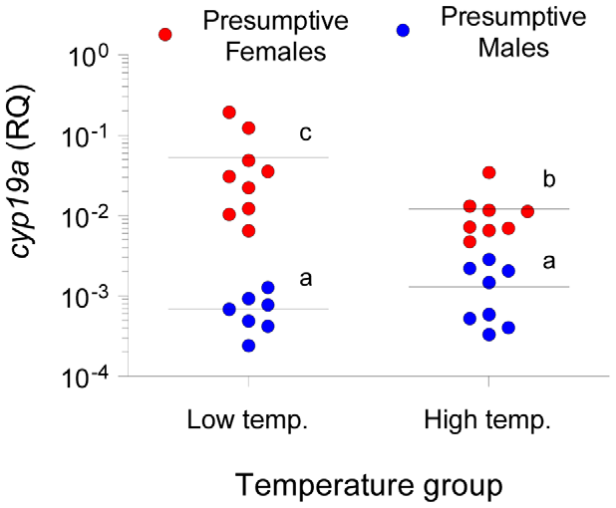
Gonadal aromatase (*cyp19a*) expression in sexually undifferentiated sea bass. Gonadal aromatase expression was assessed by real time RT-PCR and used as a marker to classify fish into presumptive females (red circles) or presumptive males (blue circles) for the two thermal treatments, low and high temperature. Individual sample points are presented along with the median. Groups with different letters are significantly different (*P*<0.05).

To examine the origin of these sex- and temperature-related differences, DNA methylation levels of the gonadal sb *cyp19a* promoter were assessed in much younger fish (94.8±0.08 mm) of an unrelated batch (family 2), in which biopsy confirmed that they were not sexually differentiated (sex differentiation in the European sea bass starts when fish are in the range ∼80–120 mm). The same temperature treatments were applied as explained above (high temperature, HT group and low temperature, LT group; see Materials and Methods and Figure S3). Since phenotypic sex was unknown in those individuals, a two-step unrestricted cluster analysis of *cyp19a* mRNA levels was used to classify fish as presumptive future males (low *cyp19a* mRNA levels) and presumptive future females (high *cyp19a* mRNA levels) within each temperature treatment (Figure 3), a procedure we had previously demonstrated to be reliable [21]. A two-way ANOVA analysis indicated significant differences in average sb *cyp19a* gene expression according to sex (F = 110.1; *P* = 0.000) but not to temperature treatment (F = 1.2; *P* = 0.277), but with interaction between the two variables (*P* = 0.013). Two-tailed *t*-tests also showed differences between *cyp19a* expression levels (RQ) of LT males vs. LT females (t = 8.427; *P* = 0.000), HT males vs. HT females (t = 6.251; *P* = 0.000), HT females vs. LT females (t = −2.516; *P* = 0.024), but no differences between the RQ values of HT males vs. LT males (P = 0.243) (Figure 3). Furthermore, methylation levels were also examined in 12 individuals who happened to be classified as presumptive females by the cluster analysis. In the HT group, *cyp19a* promoter DNA methylation levels were 79.0±3.43% (mean ± SEM, n = 3), with a coefficient of variation of 7.5% and a range of 77.2–85.7%. In the LT group, sb *cyp19a* promoter DNA methylation levels were 80.6±2.55% (two-tailed *t*-test, t = −0.373, *P* = 0.717), with a coefficient of variation of 9.5%. However, in one of the nine fish examined the value of *cyp19a* promoter methylation was 61.9%, i.e., below the threshold level of 67% calculated with the 95% confidence interval of DNA methylation levels observed in adult males vs. adult females (Figure S4), further suggesting that this fish was most likely a female.

### Sex and temperature differences in methylation levels of sb *cyp19a* promoter are tissue- and gene-specific

**Figure 4:**
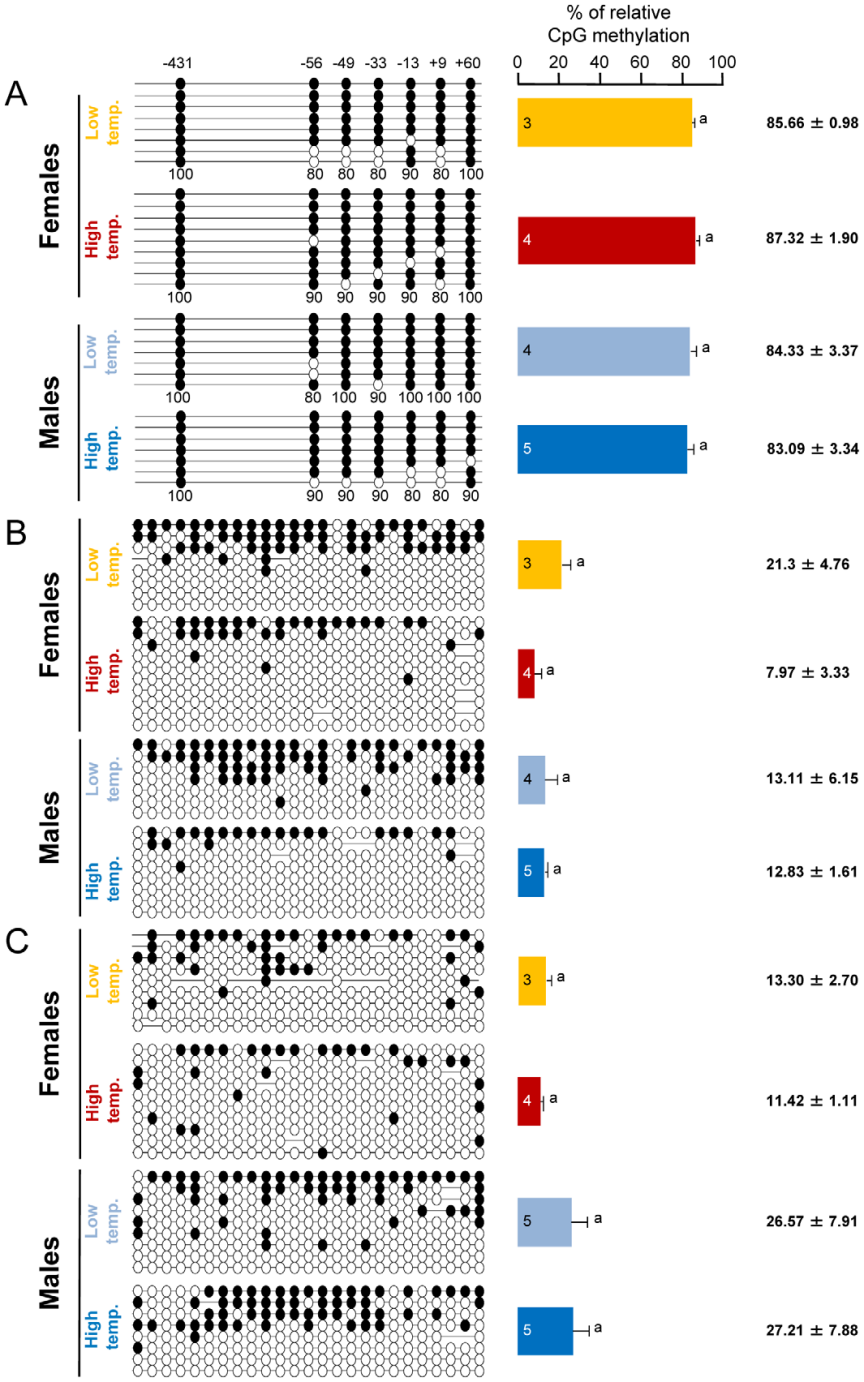
Tissue and gene specificity of the effects of temperature on DNA methylation in one-year-old sea bass. A, Resulting differences in sb *cyp19a* promoter DNA methylation in the brain according to sex and temperature treatment. B, Resulting differences in sb *ß-actin* promoter DNA methylation in the gonads according to sex and temperature treatment. C, Resulting differences in sb *ß-actin* promoter DNA methylation in the brain according to sex and temperature treatment. On the left, one fish representative of the level of methylation is shown per each sex and temperature combination. Open and filled circles denote unmethylated and methylated positions, respectively, while no circles denote unknown methylation status due to sequencing problems. Numbers with a plus or minus sign indicate CpG positions with respect to the transcription starting site. Average methylation was calculated for each position in each gene promoter (7 and 25 CpGs in the sb *cyp19a* and *ß-actin* gene promoters, respectively) but due to space limitations is shown only for the sb *cyp19a* promoter as numbers below each column (A). For each sex and temperature combination, 3–5 fish sampled at 330 dpf were used, and for each fish typically 8 clones (range 7–10) were used to determine DNA methylation levels. The number inside the bar indicates sample size. Results as mean ± SEM. Groups with the same letters are not significantly different (*P*>0.05).

In the brain, where *cyp19a* is only basally expressed, sb *cyp19a* promoter average methylation levels in one-year-old sea bass (family 1) were 85.1±0.91%, regardless of sex and/or temperature treatment. A two-way ANOVA showed lack of differences associated either with sex (F = 0.746; *P* = 0.405) or temperature (F = 0.013; *P* = 0.910) without significant interaction between the two factors (F = 0.307; *P* = 0.590) (Figure 4A). Furthermore, a similar analysis of DNA methylation of the promoter of the housekeeping gene *β-actin* in gonads of the same fish showed lack of differences associated either with sex (F = 0.191; *P* = 0.670) or temperature (F = 3.474; *P* = 0.087) without significant interaction between the two factors (F = 13.856; *P* = 0.603) (Figure 4B). Likewise, DNA methylation of the promoter of *β-actin* in the brain showed also lack of differences associated either with sex (F = 3.945; *P* = 0.068) or temperature (F = 0.038; *P* = 0.848) without significant interaction between the two factors (F = 0.029; *P* = 0.867) (Figure 4C). On average, gonad and brain *β-actin* promoter methylation levels (± SEM) were 13.8±2.76% and 19.63±4.21%, respectively.

### High temperatures experienced by the European sea bass during early life are able to masculinize populations by decreasing *cyp19a* mRNA expression levels in the female gonads

**Figure 5:**
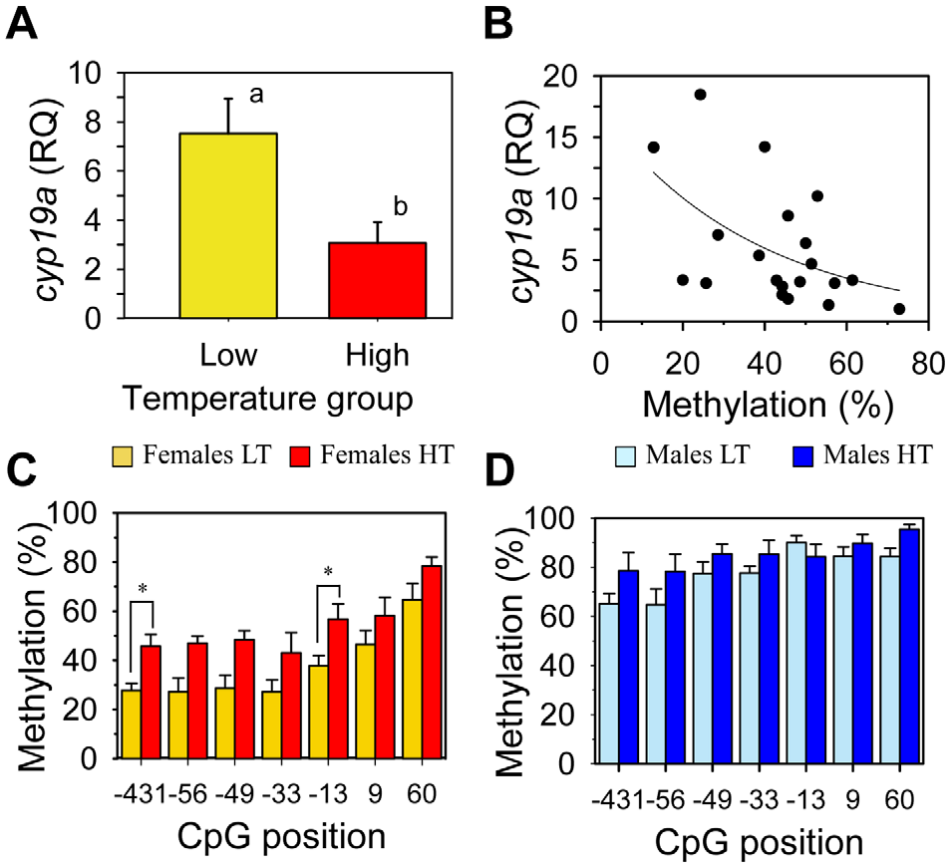
Effects of temperature on sb *cyp19a* promoter methylation levels and correlation with gonadal *cyp19a* gene expression. A, Female sb *cyp19a* expression assessed by Q-PCR; data as mean ± S.E.M (see main text for details). Groups with different letters are significantly different (*P*<0.01). B, Correlation between sb *cyp19a* methylation level and gene expression in females (male expression levels were too low to be considered). RQ, relative quantity. C and D, Differential methylation levels for each CpG position in females (C) and males (D) reared at low (LT) and high (HT) temperature; data as mean ± S.E.M. Individual CpG analysis was carried out by using Analysis of Molecular Variance (AMOVA), in which Cs were considered methylated positions and Ts unmethylated ones. Results of this analysis are summarized in Table 1.

When histologically sexed at about one year of age, the percentage of females in the HT group (family 1) was 56.0±11.3% (n = 40), a 15% decrease when compared to the 71.0±3.5% (n = 40) value of the LT group (Chi square = 7.28; *P* = 0.01). On the other hand, *cyp19a* mRNA expression levels in HT females was significantly lower than in LT females (*t*-test; F = 0.024; *P* = 0.003; Figure 5A). In addition, there was a significant inverse relationship between *cyp19a* expression and methylation levels in these one-year old females (r^2^ = 0.29; F = 7.84; *P* = 0.01) (Figure 5B).

### Administration of exogenous estrogens does not alter methylation levels of the sb *cyp19a* promoter

**Figure 6:**
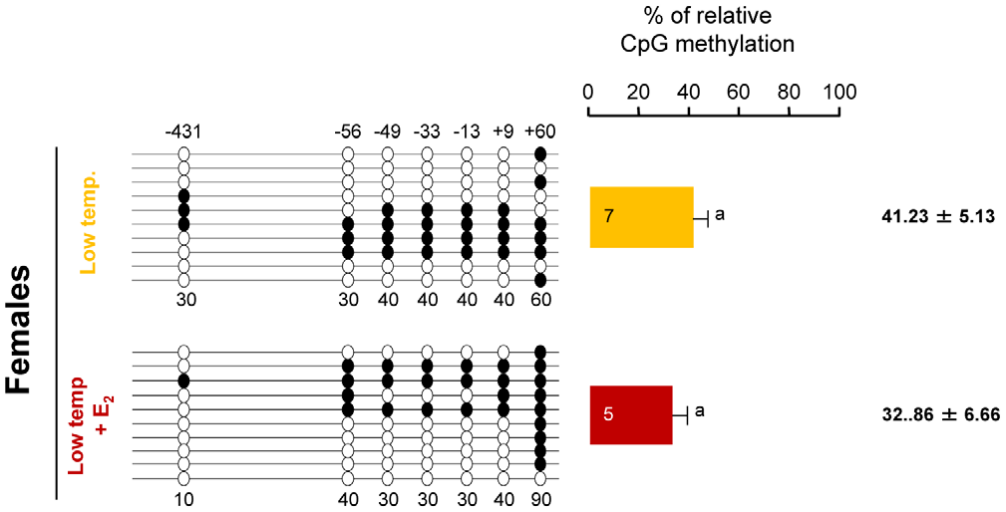
Effects of estradiol-17ß treatment on sb *cyp19a* promoter methylation in one-year-old sea bass. Result of sb *cyp19a* promoter methylation on untreated and treated females reared at low temperature. One fish representative of the level of methylation is shown for the treatment with or without estradiol-17ß (E_2_). Numbers with a plus or minus sign indicate CpG positions with respect to the transcription starting site. Open and filled circles denote unmethylated and methylated positions, respectively. Ten clones per fish were analyzed. Average methylation was calculated specifically for each position (number below each column). The number inside the bar indicates sample size. Results as mean ± SEM. Groups with the same letter are not significantly different (*P*>0.05).

We also conducted experiments to determine the possible influences of male vs. female differentiation pathways in the DNA methylation levels of the gonadal sb *cyp19a* promoter. When fish from a batch with a natural low incidence of females at LT (family 3) was treated with Estradiol-17ß (E_2_) the number of females increased from 2.5 to 90% (*P*<0.01). However, DNA methylation levels of the sb *cyp19a* promoter in the E_2_-treated female gonads were not statistically different from those of untreated LT females (mean ± S.E.M.: 32.9±6.66% vs. 41.2±5.13%; two-tailed *t*-test; t = 0.968, *P* = 0.356; Figure 6).

### Methylation levels of specific CpGs within the sb *cyp19a* promoter are sex- and temperature-dependent

Analysis of Molecular Variance (AMOVA) was used to determine if the different CpGs positions found in the sb *cyp19a* promoter were differentially methylated between male and female gonads or between the gonads of animals of the same sex but reared at different temperatures. Results revealed that particular CpGs were differentially methylated according to sex and/or temperature (Figure 5C and 5D). Significant differences in all positions were detected between LT males and LT females (AMOVA, Fst = 0.361, *P* = 0.000) (Table 1). However, only CpG positions −431 and −13 showed significant differences (AMOVA, Fst = 0.052, *P* = 0.014) when LT females were compared with HT females, with position −13 presenting the highest significant differences (Fst = 0.083, *P* = 0.031 and Fst = 0.148, *P* = 0.005, respectively; Figure 5C and Table 1).

**Table 1:** Effects of high temperature on DNA methylation of the sea bass gonadal aromatase promoter at different loci.

	LT Males versus LT Females		LT Females versus HT Females	
CpG position	Fst	*P*	Fst	*P*
−431	0.362	**0.000**	0.083	**0.031**
−56	0.219	**0.000**	−0.007	0.535
−49	0.404	**0.000**	0.009	0.325
−33	0.363	**0.000**	0.061	0.058
−13	0.575	**0.000**	0.148	**0.005**
+9	0.318	**0.000**	0.019	0.241
+60	0.168	**0.005**	0.045	0.104

For each CpG, differences between males and females exposed at low temperature (LT), and between females reared at LT and high temperature (HT) are reported. Statistically significant differences are highlighted in bold face.

Abbreviations: Fst, Fixation index of population differentiation; *P*, significance level.

### Methylation of the sb *cyp19a* promoter blocks SF-1 and Foxl2 stimulated *cyp19a* expression *in vitro*

**Figure 7:**
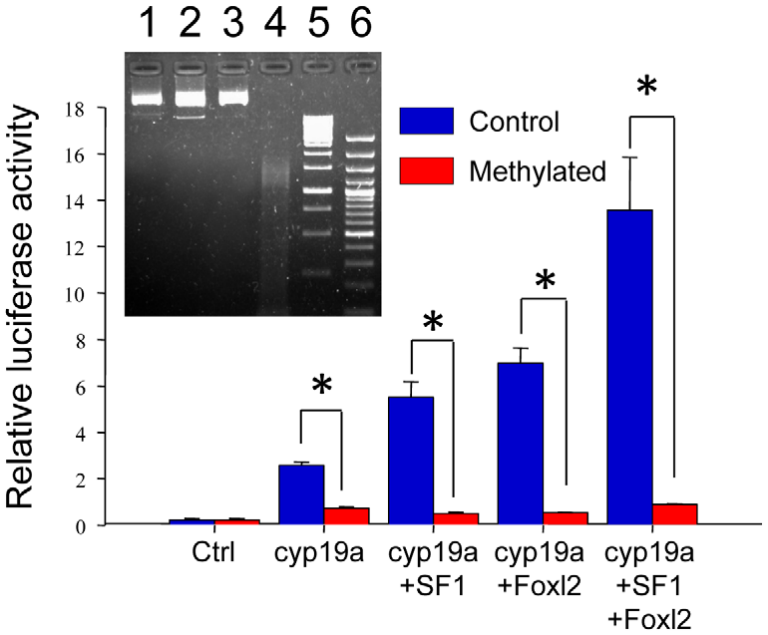
Effects of methylation on sea bass *cyp19a* promoter activity *in vitro*. HEK293T cells were transfected with pGL3-*cyp19a* methylated and unmethylated promoter vectors. Transcription factors SF1 and Foxl2 were cotransfected with the sb *cyp19a* promoter to activate promoter luciferase activity. Transfected methylated and unmethylated groups were as follows (see Materials and Methods for amounts used): 1) plasmid β-galactosidase (Ctrl); 2) sb *cyp19a* promoter cloned into pGL3-basic luciferase reporter plasmid (cyp19a); 3) sb *cyp19a* and tilapia SF1 cloned into pCDNA3.1 expression plasmid (cyp19a+SF1); 4) sb *cyp19a* and tilapia Foxl2 cloned into pCDNA3.1 expression plasmid (cyp19a+Foxl2); 5) sb *cyp19a*, tilapia SF1 and Foxl2 cloned into pCDNA3.1 (cyp19a+SF1+Foxl2). The Student's *t*-test was used to compare methylated and unmethylated vectors. Significant differences are denoted by an asterisk and were as follows: cyp19a, *P* = 0.006; cyp19a+SF1, *P* = 0.006; cyp19a+Foxl2, *P* = 0.003; cyp19a+SF1+Foxl2, *P* = 0.013. Also, SF1 and Foxl2 exhibited an additive effect since the activation of sb *cyp19a* promoter was significantly higher when both were transfected together. Values are shown as mean ± S.E.M. (n = 2–4). Insert: Successful vector methylation verification by analysis of band patterns on electrophoresis gel after digestion of the purified plasmids with the *McrBC* enzyme. Lane 1, 0.5 µg sb *cyp19a*-pGL3; lane 2, 0.5 µg sb *cyp19a*-pGL3 treated with *McrBC*; lane 3, 0.5 µg *Sss*I-methylated sb *cyp19a*-pGL3; lane 4, 0.5 µg *Sss*I-methylated sb *cyp19a*-pGL3 treated with *McrBC*; lane 5, 1 Kb marker; lane 6, 100 bp marker. The electrophoresis gel shows that, as expected, only the methylated vector was digested.

In a previous study [27], we characterized the sb *cyp19a* promoter using bioinformatic tools (MatInspector) and gel shift assays and identified putative transcription binding sites. From that study it was found that the CpG in position −431 of this promoter is located near a putative binding site for a transcription factor of the Fox family and that the CpG in position −13 is located near a putative binding site for a Sox family transcription factor (Figure S2). Furthermore, previous studies with other fish species have shown that co-transfection of sb *cyp19a* promoter constructs with either Foxl2, SF-1 or simultaneous co-transfection with both of these potent transcriptional activators of *cyp19a* significantly increased luciferase activity [28], [29]. Based on these previous findings, the sb *cyp19a* promoter activation was determined under methylated and control conditions by a luciferase reporter assay. As expected, SF-1 and Foxl2 were each capable of activating sb *cyp19a* expression *in vitro*. When combined, still higher expression was observed (Figure 7). Remarkably, induced hypermethylation of the sb *cyp19a* promoter completely suppressed promoter transcription stimulation *in vitro* by Foxl2 and SF-1 alone (two tailed *t*- test, *P*<0.01) or in combination (two tailed *t*- test, *P*<0.05).

### Specific CpG positions of the *cyp19a* promoter are conserved in other species

**Figure 8:**
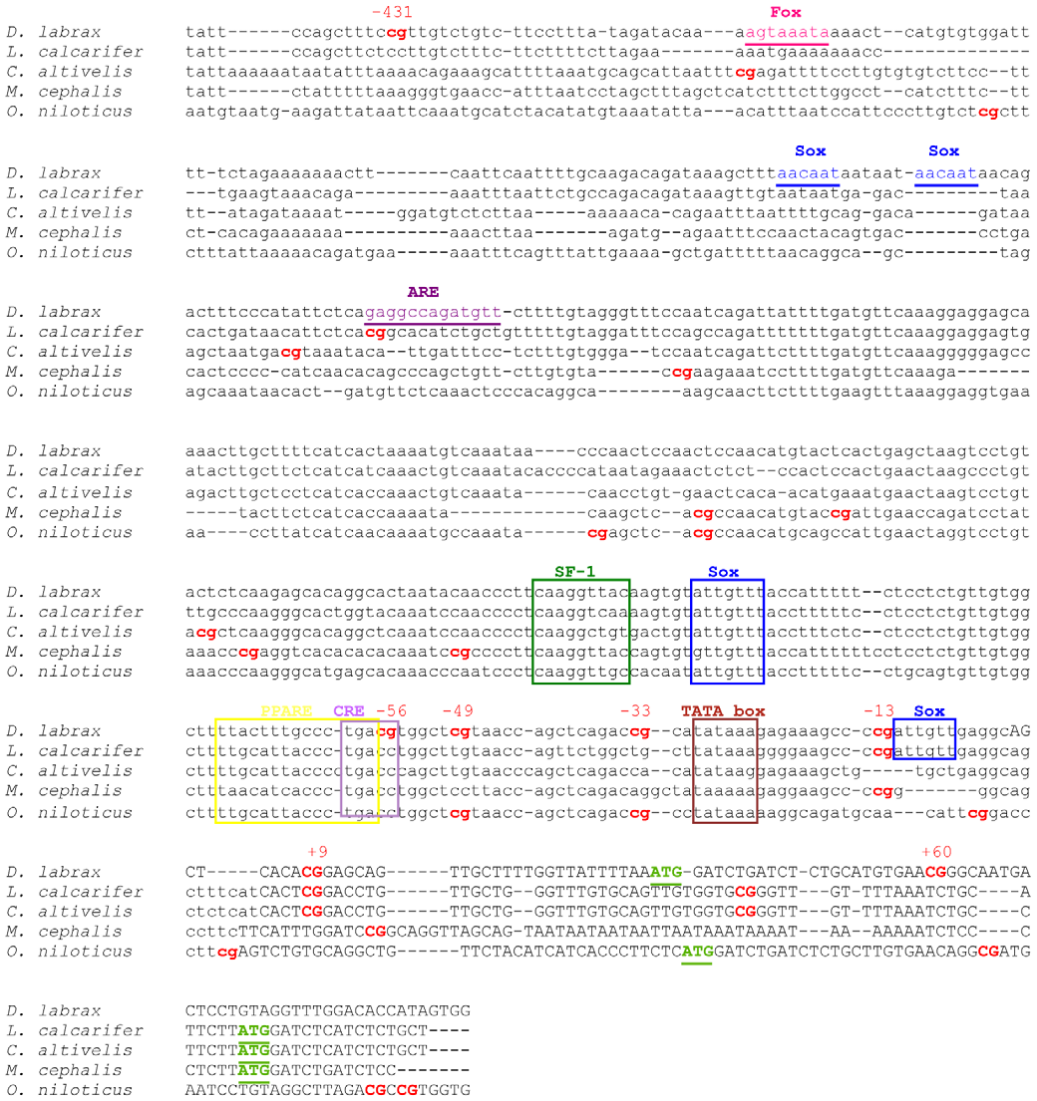
Alignment of the sea bass gonadal *cyp19a* promoter with that of other teleost species. Accession numbers: *Dicentrarchus labrax*, DQ177458; *Lates calcarifer*, AY686690; *Cromileptes altivelis*, AY686691; *Mugil cephalus*, AY859426; *Oerochromis niloticus*, AB089924. CpG dinucleotides are shown in bold. The numbers above *D. labrax* CpGs denote the position relative to the transcription start site. Putative binding sites for specific transcription factors were localized in *D. labrax* after Galay-Burgos *et al.* [6] and are indicated by boxes when are conserved among species or by underlying if apply only to *D. labrax*. The open reading frame is depicted by upper case text and putative transcription start sites “ATG” are highlighted in bold and underlined.

To determine whether the number and position of CpGs was conserved in other fish species, 600 bp of the sb *cyp19a* promoter, including all CpG analyzed in the present study and 94 bp within the opening reading frame, were aligned with the promoter region of both phylogenetically-related and unrelated species. In addition and based on the previously characterization of the sb *cyp19a* promoter by Galay-Burgos and collaborators [27] some putative transcription binding sites for other species were identified based on sequence similarity with the sb *cyp19a* promoter. Overall sequence similarity was 53%, with a clear conservation of some of the transcription factor binding sites, including the TATA box (Figure 8). The CpG located at position −13 was the most conserved, with three species having a CpG dinucleotide at this position. On the other hand, position −431 was not found in the other species analyzed although two of them had a CpG position situated around 40–60 bp away.

## Discussion

### Methylation levels of the sb *cyp19a* promoter are significantly higher in males than in females

The present study clearly shows that the DNA methylation levels of the sb *cyp19a* promoter were twice as high in the gonads of males as compared to females in one-year-old European sea bass. The origin of these sex-related differences is at present unknown. Throughout development, cell- and tissue-specific methylation patterns are the result of *de novo* methylation, maintenance of the existing pattern and demethylation processes [30]. It has been suggested that DNA methylation suppresses recombination, allowing Muller's ratchet to operate on differentially methylated sexes [10]. Differential methylation can also suppress transcription, contributing to accentuate sex-related differences [10]. Thus, sex differentiation of the gonads could be regulated through DNA methylation of key genes in a manner similar to that seen in other tissues. SRY, the sex-determining gene in mammals, is normally epigenetically silenced, activated during a specific window in development, and then silenced again [31]. The machinery needed for these changes is also present in fish, where up to eight different DNA methyltransferases (Dnmts), the enzymes responsible to transfer methyl groups to DNA, have been identified so far [32]. In medaka (*Oryzias latipes*) and *Xiphophorus* embryos, dnmt1 expression is spatially- and temporally-regulated, suggesting that it may play an important role during development. Changes in methylation levels of *cyp19a* and other gene promoters are then likely to be involved in the course of sexual differentiation in fish.

To elucidate the possible influences of male vs. female differentiation pathways, we measured DNA methylation levels of the sb *cyp19a* promoter in gonads of E_2_-treated vs. control females (both reared at LT to avoid confounding effects of masculinization by HT). No differences were found, indicating that E_2_ in this model does not affect gonadal sb *cyp19a* promoter DNA methylation levels. This is consistent with previous studies showing that *cyp19a* expression levels in gonads of both untreated and E_2_-treated females were similar [33]. Also, treatment of medaka with estrogen did not result in changes *cyp19a* methylation in the gonads [34]. In the present study, the lack of effects of estrogen treatment on gonadal sb *cyp19a* promoter methylation levels supports the idea that increased methylation is the cause of lower *cyp19a* expression and not the other way around.

### High temperatures experienced during early life in the European sea bass are able to masculinize populations by increasing gonadal sb *cyp19a* promoter DNA methylation and decreasing *cyp19a* mRNA expression

Temperature effects on the methylation of certain promoters are well established in plants [35]. There is also evidence of environmental influences on phenotypic plasticity in animals mediated by epigenetic mechanisms [36]. In honeybees, nutrition load gives rise to two different adult female phenotypes: the fertile queens and the sterile workers [37]. Recently, it has been shown that the active ingredient in royal jelly involves an epidermal growth factor receptor-mediated signaling pathway, resulting in increased body size, ovarian development and shortened developmental time [38]. Interestingly, in many fishes including the sea bass, there is also an association between larger size and female development during sex differentiation [39], [40].

The European sea bass has a polygenic system of sex determination [18] with a strong environmental influence [4]. Sex ratios depend upon the broodstock used and the temperature experienced during the TSP [20]–[25]. Because of its sex determining mechanism, sea bass monosex populations are not available. To determine the influence of temperature in the methylation levels of the gonadal sb *cyp19a* promoter, we had deliberately chosen a family giving a high percentage of females when reared at the permissive LT (family 1). This allowed obtaining sufficient females even after rearing at masculinizing HT. The LT or control group had 71±3.5% females, whereas the HT group had 56±11.3% females, in agreement with the observation that rearing at HT during the TSP masculinizes ∼50% of the genotypic females into phenotypic males without negative effects on survival [20].

The main finding of this study is that exposure to high temperature during early development increased ∼1.5 times sb *cyp19a* promoter DNA methylation levels as evidenced in one-year-old female gonads. In contrast, HT did not significantly increase sb *cyp19a* promoter methylation levels in the gonads of males, which were already quite high (∼80%) in the LT group. In the present study, also a relationship between increased methylation and decreased female numbers was found since the reduction of 15% females in the HT group closely matched the 16.8% increase in sb *cyp19a* promoter methylation levels in the same group. Importantly, *cyp19a* expression was significantly lower in HT females. Thus, the present study demonstrates that high temperatures experienced during early life masculinize by increasing sb *cyp19a* promoter methylation levels and consequently decreasing *cyp19a* expression in the gonads. It could be argued that differential methylation of the sb *cyp19a* promoter could be part of the polygenic mode of sex determination by parental imprinting mechanisms. However, the existence of differences in methylation levels of the sb *cyp19a* promoter in the gonads of LT and HT females (originated from the same parents) suggests that high temperatures is able to override any possible parental imprinting. This indicates that the male-biased sex ratios found in stocks reared at HT is a temperature effect overriding the basic polygenic sex determination system.

Since genes that are not in use may become methylated, it could be argued that *cyp19a* suppression is the consequence rather than the cause of the suppression of female development, i.e., that methylation of the sb *cyp19a* promoter can be due to the initiation of the male pathway. Our results with sexually undifferentiated animals show differences on gonadal *cyp19a* gene expression levels between presumptive males and females, in agreement with the observation that differences in gonadal *cyp19a* gene expression levels can be detected prior to sex differentiation [41]. Methylation levels of the gonadal sb *cyp19a* promoter on presumptive females reared at LT or HT ranged from 61.9 to 83.7%. Interestingly, these values are closer to those of one-year-old males (range 71.4–97.1%) than those of one-year-old females (range 12.9–72.8%). This suggests that the methylation levels in presumptive females may reflect low gonadal *cyp19a* gene expression levels at that time of development. Further, although differences between putative females and males can be detected prior to sex differentiation [41], upregulation of *cyp19a* gene expression only occurs during female sex differentiation [39]. Together, these observations suggest that during fish sex differentiation DNA demethylation of the sb *cyp19a* promoter in the females could be required to enable *cyp19a* upregulation in their gonads. During mammalian gonadal development, genome-wide demethylation occurs during primordial germ cell migration in early development as observed in mice and pigs [42]–[44]. We hypothesize that high temperature during early development either immediately and irreversibly hypermethylates the *cyp19a* promoter or inhibits its demethylation later during sex differentiation. Further studies are needed to discern between these two possibilities.

We also checked whether the sex-and temperature-dependent changes on sb *cyp19a* promoter methylation in the gonads were promoter- and tissue-specific. It is known that the promoters of non-expressed genes are hypermethylated. The sb *cyp19a* promoter in the brain was consistently hypermethylated independent of sex and temperature, in accordance with the well-established fact that *cyp19a* is mainly expressed in gonads and only basally expressed in the fish brain [45]. In contrast, the *β-actin* promoter was hypomethylated both in brain and gonads and regardless of sex and temperature, reflecting the constitutively high expression of this housekeeping gene. Together, these results suggest that in the European sea bass sex- and temperature-dependent changes in DNA methylation of the *cyp19a* promoter are restricted to the gonads and are not a generalized effect of temperature, although other gene promoters could also be affected.

The mechanism by which temperature changes DNA methylation levels of the sb *cyp19a* promoter in the gonads is not known. In *Xiphophorus*, O^6^-methylguanine-dnmt activity is optimal at 23°C but is lost below 15°C [46]. Thus, it remains to be determined if temperature activation of the Dnmts is responsible for the resulting higher *cyp19a* promoter methylation seen at HT. Since *cyp19a* expression is essential for female differentiation in all non-mammalian vertebrates, resolving the link between temperature and *cyp19a* promoter methylation is a relevant issue. The results presented here, although mostly descriptive, represent a first step in this direction.

### Effects of DNA methylation on specific CpG positions of the gonadal sb *cyp19a* promoter may be conserved in other fish species

Foxl2 is one of the most potent transcriptional regulators of *cyp19a* in vertebrates, from fish to mammals [28], [47]; however, it appears that Foxl2 works best with the involvement of other cofactors. For example, co-transfection of *cyp19a* promoter constructs with either SF-1 or Foxl2 significantly increased luciferase activity [28], but simultaneous co-transfection of SF-1 and Foxl2 had an additive effect on *cyp19a* promoter activation [29]. The present study shows, as expected, that SF-1 and Foxl2 were each capable of activating *cyp19a* expression in HEK293T cultured cells, and when combined still higher expression was observed. Remarkably, induced hypermethylation of the sb *cyp19a* promoter completely suppressed both Foxl2- and/or SF-1-stimulated transcription *in vitro*. This strongly suggests that hypermethylation of the sb *cyp19a* promoter prevents binding of at least Foxl2 and SF-1 to their respective sites, thus blocking *cyp19a* transcriptional activation.

Sex- and tissue-specific differences on *cyp19a* methylation levels have also been found in the model fish medaka, a GSD species, where five CpGs located within a region of ∼300 bp of the *cyp19a* promoter were methylated mostly in testis and female brain, but unmethylated in ovary and male brain [34]. Further, in cattle and sheep *cyp19a* promoter regions P1.1, P1.5 and P2 were differentially methylated depending on sex and tissue [15]. Together, these results suggest that sex-related differences in DNA methylation levels of the *cyp19a* promoter maybe a generalized phenomenon present from fish to mammals. On the other hand, it has been suggested that the molecular mechanisms underlying sex ratio responses to temperature must be conserved throughout vertebrates [1], [8], [48]. Our results revealed that some of the transcription factors binding sites in the *cyp19a* promoter are conserved across species, being the CpG dinucleotide located at position −13 the most conserved one. Together, these observations suggest that the epigenetic mechanism described here may indeed be present at least in other fish species. Further, this study also shows that some sb *cyp19a* promoter CpG positions are differentially methylated by temperature, indicating that they can be important for *cyp19a* transcription. This is also the case of position −13, near a TATA box and Sox binding site, or position −431, which is close to a Fox binding site. Differential DNA methylation of gene promoters has been demonstrated to account for tissue-specific gene transcription through transcription factor binding inhibition [49]–[51]. Together the results presented here suggest that differential methylation of specific CpGs positions could significantly contribute to a transcription regulation of the sb *cyp19a* gene.

**Figure 9:**
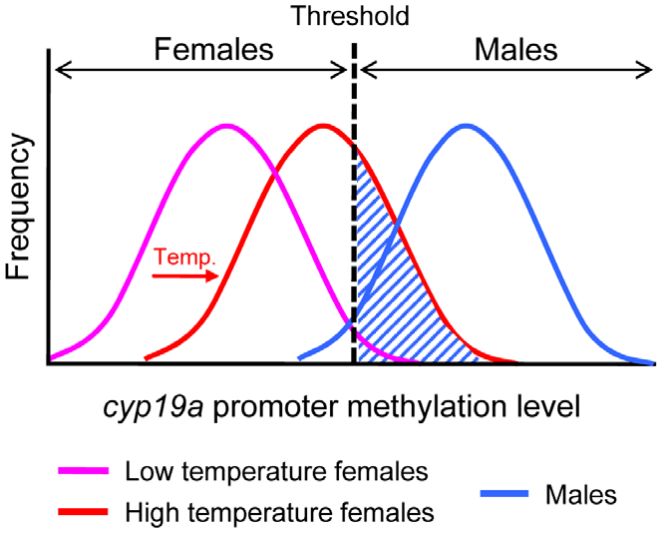
Schematic diagram of the frequency distribution of the sb c*yp19a* promoter methylation levels in females and males. Low methylation levels (purple curve) of the sb *cyp19a* promoter are typical of females and are required for ovarian differentiation. Males typically show high methylation levels (blue curve) of the *cyp19a* promoter. This blocks aromatase transcription and induces testicular differentiation caused by lack of estrogens. As shown in this study, high temperatures (HT) during the thermosensitive period increase methylation levels of the sb *cyp19a* promoter (red curve), shifting the female distribution closer to that of males. The dashed area represents the fraction of genotypic females that are sex reversed into phenotypic males by high temperature.

In summary, to our knowledge, this is the first report describing an epigenetic mechanism mediating temperature effects on sex ratios in any animal. Our data show that the sb *cyp19a* promoter is significantly more methylated in males than females, which is in agreement with the well-established constitutively lower levels of *cyp19a* expression in testes as compared to ovaries, and with the higher levels of estrogens seen in females [6]. Further, luciferase assays confirmed that DNA methylation of the sb *cyp19a* promoter represses transcription *in vitro*. Together, these results indicate that hypomethylation of the *cyp19a* promoter is required for normal ovarian development. Further, since estrogens are essential for ovarian differentiation, our results suggest that sb *cyp19a* promoter hypermethylation contributes to *cyp19a* transcription silencing during male differentiation, preventing the transformation of an undifferentiated gonad into an ovary. More importantly, however, we show that in a fish species where sex determination depends on the interaction between genotype and environment, exposure to abnormally high temperatures during the TSP is able to induce hypermethylation of the sb *cyp19a* promoter of females past a certain threshold, approaching the values characteristic of males (Figure 9). The result is that genotypic females—or, more properly in a mixed genic and environmental sex determination system, fish in which the sum of factors promoting female development is stronger than the sum of factors promoting male development— differentiate into phenotypic males, altering population sex ratios, as observed in many fish species exposed to high temperatures [4], [6]. As shown by Ospina-Álvarez and Piferrer [4], and in contrast to reptiles [1], fish studied so far have only one sex ratio response pattern to temperature: male-biased sex ratios with increased temperatures. This makes still more appealing the methylation hypothesis because the underlying mechanism could apply to all fish species with temperature influences on sex ratios. Importantly, it has been suggested that in species with GSD such as mammals, where sex determination depends on the inheritance of the sex-determining gene SRY, sex is a threshold dichotomy mimicking a single gene effect [52]. Our results indicate that such a threshold dichotomy can also apply to a completely distinct scenario: gonadal *cyp19a* promoter methylation levels of males vs. females, implying one shared feature of the two major sex determining mechanisms of vertebrates, GSD and TSD. Thus, temperature may modulate sex ratios through changes in the proportion of animals whose *cyp19a* promoter methylation level falls above or below a certain threshold. The present results demonstrate that in the European sea bass an epigenetic mechanism can affect an essential biological process, with consequences in resulting population sex ratios. Although more studies are certainly needed to confirm this, it is tempting to suggest that the epigenetic scheme described herein may be an essential component of the long-sought-after mechanism connecting environmental temperature and sex ratios in species with TSD.

## Materials and Methods

### Animal rearing conditions and treatments

Freshly fertilized European sea bass (*Dicentrarchus labrax* L.) eggs were collected from the Institute of Aquaculture (Castelló, Spain) and from a private farm (Base Viva, St. Pere Pescador, Spain) and transported to our experimental aquarium facilities at the Institute of Marine Sciences in Barcelona. Egg incubation and rearing during the larval and juvenile stages were performed according to standard sea bass rearing practices [53], except for the temperature treatments (see next section), and are explained in detail elsewhere [20]. Once animals reached mid-metamorphosis (standard length; SL>18 mm), juveniles were reared in 650 l fiberglass tanks under simulated natural photoperiod and fed *ad libitum* with pelleted food of the appropriate size.

#### Temperature treatments

Eggs were incubated at 14–15°C, the natural temperature for sea bass spawning and fertilization during winter and early spring in the western Mediterranean. For this study, a total of 3 different egg batches originated from different parents (i.e., 3 different families) were used. Hatching occurred 3–4 days post fertilization (dpf). At this point, fish were separated into two groups. One group was reared at 15°C throughout the thermosensitive period (TSP) until 60 dpf (“low temperature”, LT or control group). Then, temperature was increased to 21°C at a rate of 0.5°C·day^−1^ and left to follow the natural fluctuations until the end of the study, when fish were about one year old (330 dpf). Twenty-one degrees Celsius is the standard temperature for ensuring adequate growth in juvenile sea bass rearing. The other group of fish was reared at 15°C until 10 dpf and then switched to 21°C (Figure S3). Thus, fish in this group were exposed to artificially higher temperature (HT) essentially during the entire TSP, which promotes male development. Each temperature treatment was carried out in duplicate. In summary, while the thermal regimen of the LT group is similar to the one experienced by sea bass in the wild, the thermal regimen of the HT group typically results in the masculinization of about one-half of the genotypic females into phenotypic males [19], [20]. For these experiments, two different sea bass families (families 1 and 2) were used: sb *cyp19a* and *β-actin* promoter methylation was studied in brain and gonads and *cyp19a* gene expression in gonads of sexually differentiated, one-year-old animals (family 1), and sb *cyp19a* promoter methylation and gene expression was studied in the gonads of sexually undifferentiated animals (family 2).

#### Estrogen treatments

An additional group of fish subjected to the same LT regime as described above was fed with a diet containing estradiol-17β (E_2_) at a concentration of 10 mg/kg of food. Treatment was carried out well past the TSP, from 90–150 dpf, coinciding with the labile period for steroid treatment [19]. This experiment was carried out to test whether E_2_ treatment was capable of affecting sb *cyp19a* promoter methylation, and hence, in order to maximize the expected feminizing effect of E_2_, in this case a family giving low numbers of females even at LT was used (family 3). At about one year of age, fish (n = 40) were sexed and DNA methylation levels were determined as explained below.

#### Ethics statement

In all cases, fish were treated in agreement with the European Convention for the Protection of Animals used for Experimental and Scientific Purposes (ETS N° 123, 01/01/91).

### Sampling and gonadal histology

At 330 dpf, i.e., long past the thermal regimes, fish were sacrificed and gonadal samples were collected (n = 40 fish per treatment). From each fish (∼159 mm and ∼73 g), one gonad was processed for histological identification of sex. Gonads were fixed in 4% paraformaldehyde in PBS, embedded in paraffin, cut at 7 µm thickness and stained with haematoxylin-eosin. The other gonad was snap-frozen in liquid nitrogen and stored at −80°C until further analysis to determine methylation levels and gene expression. For sexually undifferentiated fish (family 2; 94.8±0.08 mm), 20 fish per group were collected from the LT and HT treatment groups. Due to tissue amount limitations, the right gonad was used to obtain DNA and the left gonad to obtain RNA. Sex was identified based on *cyp19a* mRNA levels as described in Blázquez and collaborators [41] and in the Statistical analysis section below.

### Methylation levels measured by bisulphite-mediated genomic sequencing

The gonadal sb *cyp19a* [27] and *β-actin* promoters [54] were examined to identify CpG dinucleotides that could be differentially methylated. For the sb *cyp19a* promoter (Figure S2), genomic DNA was obtained in the case of sexually differentiated fish from the gonads of 8–15 fish or the brains of 3–5 fish, depending on temperature and sex. For the *β-actin* promoter, brains and gonads from 3–5 animals were used depending also on temperature and sex. In the case of sexually undifferentiated fish, a total of 12 animals were used for sb *cyp19a* promoter methylation analysis. The DNA samples from each animal were individually processed and subjected to sodium bisulphite-mediated sequencing as described by Widschwendter and collaborators (2000) [55]. The targeted portion of the promoter was amplified from the bisulphite-modified DNA with two rounds of PCR by use of nested primers specific to the bisulphite-modified sequence of this region. For the sb *cyp19a* promoter the primers were as follows: External Forward, ATTGGTAGTTTAATGGAGGAATTT; External Reverse, AATCCCACTACAATAACATTTAAAAAC; Nested Forward, GAGGAATTTGGGAGGAATTATAAATAT; Nested Reversed, CCAAATCTACCACTATAATATCCAAAC. The primers for the *β-actin* promoter were as follows: External Forward, AATTTATAATTTTGGTTGGTAGTAA; External Reverse,CAAAATCTTACCTTAAAAATATATCTAC; Nested Forward, TATAATTTTGGTTGGTAGTAATTGG; Nested Reverse, CATTCACAAACCTCAACACTAACC. A hot start polymerase (Qiagen) was used in both external and nested PCR. For the sb *cyp19a* promoter, external PCR consisted in 5 min at 94°C; 5 cycles of 1 min at 94°C, 2 min at 55°C, 3 min at 72°C; 25 cycles of 30 s at 94°C, 2 min at 50°C, 1 min and 30 s at 72°C and a final extension of 7 min at 72°C. Subsequently, nested PCR consisted in 5 min at 94°C; 30 cycles of 30 s at 94°C, 30 s at 56°C, 30 s at 72°C and a final extension of 7 min at 72°C. For the *β-actin* promoter, external PCR consisted in 5 min at 94°C; 5 cycles of 1 min at 94°C, 2 min at 54°C, 3 min at 68°C; 25 cycles of 30 s at 94°C, 2 min at 50°C, 1 min and 30 s at 68°C and a final extension of 7 min at 72°C. Subsequently, nested PCR consisted in 3 min at 94°C; 30 cycles of 30 s at 94°C, 30 s at 53°C, 30 s at 68°C and a final extension of 7 min at 72°C. PCR products were separated by gel electrophoresis and gel bands were purified by Purelink Quick Gel Extraction (Invitrogen). Gel purified bands were cloned into the pCR4-TOPO vector and transformed into *E. coli* Topo10 chemically competent cells (Invitrogen) in the case of the sb *cyp19a* promoter, or into pGEM-T Easy vector (Promega) and transformed into *E. coli* JM109 competent cells (Invitrogen) in the case of the *β-actin* promoter. Then 7–10 individual clones per each fish were sequenced each in both directions and used to evaluate the seven (*cyp19a*) or 25 (*β-actin*) CpG dinucleotide positions present in the promoter region. Average methylation levels per position and fish were computed. In summary, in this study a total of 76 different animals were used. DNA methylation levels were determined, always on an individual fish basis (no pools were used), only in the gonads in some fish, whereas in others it was determined both in the gonads and also in the brain, and, as stated above, 7–10 clones per fish were sequenced in both directions. The total amount of sequenced clones in this study was ∼1100.

The efficiency of the bisulfite conversion step was evaluated by using the Bisulfite sequencing Data Presentation and Compilation (BDPC) online software (available at: http://biochem.jacobs-university.de/BDPC/index.php) [56]. The conversion rates of C, which are not in the context of a CpG, were determined in different number of clones and in all tested tissues. For the sb *cyp19a* promoter, the mean percentage of converted Cs was 97.98±0.9% (calculated in a subset of 35 PCR reactions) and for *β-actin* promoter, the mean percentage was 97.68±0.41% (calculated from a subset of 71 PCR reactions).

Since ten clones per fish were used to determine sb *cyp19a* promoter methylation, the maximum number of different methylation patterns (or epialleles) per fish that could be observed was 10, i.e., each clone has a different methylation pattern. Thus, for each fish from each one of the four groups the actual observed number of different *cyp19a* promoter methylation patterns was determined in order to check for possible PCR bias.

### *sb cyp19a* gene expression measurement by real-time RT–PCR

Total RNA was isolated from ovaries of 8 females from the HT group and 14 females from the LT group from sexually differentiated one-year-old sea bass, and 16 gonads from the HT and 16 from the LT group in the case of sexually undifferentiated or differentiating animals. Gonad tissue was homogenized with 0.5 ml of trizol and total RNA was extracted with chlorophorm, precipitated with isopropanol and washed with 75% ethanol. Pellets were suspended in 25 µl DEPC-water and stored at −80°C. One microgram of total RNA was reverse transcribed into cDNA using Superscript II (Invitrogen) and 250 ng of random hexamer primers (pdN6) following the manufacturer's instructions. Real-time PCR reactions were carried out to determine sb *cyp19a* gene expression. The endogenous reference gene used was *18S* (validated in a previous study by our laboratory [57]). The real time RT-PCR reaction was carried out with the SYBR Green chemistry (Power SYBR Green PCR Master Mix; Applied Biosystems). The primers were: ovaro RT-F1: AGACAGCAGCCCAGGAGTTG and ovaro RT-R1: TGCAGTGAAGTTGATGTCCAGTT. PCR reactions contained 1X SYBR green master mix (Applied Biosystems), 10 pmol of each primer and 1 µl of the RT reaction. Samples were run in duplicate in optically clear 384-well plates. Cycling parameters were: 50°C for 2 min, 95°C for 10 min, followed by 40 cycles of 95°C for 15 s and 60°C for 1 min. Finally, a temperature-determining dissociation step was performed at 95°C for 15 s, 60°C for 15 s and 95°C for 15 s at the end of the amplification phase. Real-time RT-PCR data were collected by SDS 2.3 and RQ Manager 1.2 software and relative quantity (RQ) values were estimated for each reaction replicate. Specifically, the female with the lowest level of aromatase expression (i.e., highest ΔCt) was assigned as the calibration sample to calculate ΔΔCt and RQ values.

### *cyp19a* promoter activation by luciferase reporter assay

#### Plasmid construction

Genomic DNA was used to clone the sb *cyp19a* promoter by PCR. The primers used were: AroALucF, GCAGAGGTAGGAACACAGTTCA and AroALucR, CATTTGGGGACGTGGAGA. To each 5′-end primer sequence a restriction site for *Xho*I enzyme was added. Promoter sequence was obtained using Easy-A High-fidelity PCR cloning polymerase (Stratagene) and PCR conditions were: 2 min at 95°C; 25 cycles of 30 s at 95°C, 30 s at 68°C, 2 min and 30 s at 72°C and a final extension of 5 min at 72°C. The amplified promoter fragment was gel purified and cloned into pGEM-T (Promega). The sb *cyp19a* promoter was subsequently digested with *Xho*I enzyme from pGEM-T vector and cloned into *Xho*I digested pGL3 Basic Vector (Promega).

#### *In vitro* methylation of plasmid reporters

pGL3-*cyp19a* plasmids were cytosine-methylated using *Sss*I methylase according to the manufacturer's instructions (New England BioLabs). *Sss*I methylation, which methylates all cytosine residues within the double-stranded dinucleotide recognition sequence (5′-CG-3′), was performed with 10 mM Tris, pH 7.9, 50 mM NaCl, 10 mM MgCl_2_, 1 mM DTT, and 160 µM S-adenosylmethionine at 37°C for 1 h. After the methylation, reaction plasmids were purified by phenol extraction. Successful vector methylation was checked by analyzing band patterns on gel electrophoresis after digestion of the purified plasmids with the *McrBC* enzyme, which digests only methylated DNA, according to the manufacturer's instructions (New England BioLabs). Fully methylated plasmids were utilized for transient transfection assays.

#### Transfection and luciferase reporter gene assay

Human embryonic kidney 293T (HEK 293T) cells were transfected using the calcium phosphate coprecipitation method [58] with pGL3-*cyp19a* methylated and unmethylated promoter vectors. Transcription factors SF1 and Foxl2 were cotransfected with the sb *cyp19a* promoter to activate promoter luciferase activity. Transfected methylated and unmethylated groups were as follows: 1) 500 ng of plasmid β-galactosidase (Ctrl); 2) 2.5 µg of sb *cyp19a* promoter cloned into pGL3-basic luciferase reporter plasmid (cyp19a); 3) 2.5 µg of cyp19a and 250 ng of tilapia SF1 transcription factor cloned into pCDNA3.1 expression plasmid (cyp19a+SF1); 4) 2.5 µg of cyp19a and 250 ng of tilapia Foxl2 transcription factor cloned into pCDNA3.1 expression plasmid (cyp19a+Foxl2); 5) 2.5 µg of cyp19a, 250 ng of tilapia SF1-pCDNA3.1 and 250 ng of tilapia Foxl2-pCDNA3.1 (cyp19a+SF1+Foxl2). Five hundred nanograms of plasmid β-galactosidase were co-transfected in all cases as an internal control of transfection efficiency. All transfections were carried out at least in duplicate. Cells were incubated for 16 h with precipitated vectors-CaPO_4_. Forty-eight hours after transfection, cells were washed with PBS and lysed in 400 µl luciferase lysis buffer. Lysate was analyzed for Luc activity using the luciferase assay system (Promega) in an Orion Microplate luminometer (Berthold). β-galactosidase activity was used to normalize the results.

### Statistical analyses

Data reported as proportions (sex ratios and methylation levels) were always arcsin square root transformed before any statistical analysis. Likewise, all RQ expression data were log-transformed to ensure normality.

A two-way ANOVA was used to investigate differences in sb *cyp19a* or *β-actin* promoter DNA methylation levels (dependent variable) considering sex and temperature as the two independent factors. *Post hoc* multiple comparisons were carried out with Tukey's multiple range test with the Statgraphics v16 or SPSS v19 software.

One-year-old fish were histologically sexed. Younger, sexually-undifferentiated fish were sexed based on a previous study from our laboratory that demonstrated that mRNA levels of *cyp19a* can be used as an early marker of phenotypic sex in the European sea bass [41]. In this case, a two-step cluster analysis (SPSS) was used to classify individuals as presumptive females and males based on the gonadal *cyp19a* mRNA levels (RQ). Afterwards, a two-way ANOVA was used to investigate differences between temperature and phenotypic sex as explained above. In addition, a two-tailed Student's *t*-test was used to check for differences in RQ between the following pairs: presumptive males and females at LT; presumptive males and females at HT; presumptive females at LT and HT; and presumptive males at LT and HT. Two-tailed Student's *t*-test was also used to analyze differential *cyp19a* expression levels among one-year old females of each temperature treatment and also to detect differences between pGL3-cyp19a methylated and unmethylated promoter vectors in the transfection assay.

To check for differences in methylation levels in specific CpG positions of the sb *cyp19a* promoter, a hierarchical population analysis was carried out. First, sequences were trimmed to seven nucleotides in length, one corresponding to each CpG analyzed, with two possible variants for each nucleotide: C if methylated and T if unmethylated. Then, all sequences from the same individual were considered as one population, with size equivalent to the number of sequences analyzed for each individual [59]. The four classes (i.e., treatments, LT and HT males, and LT and HT females) were considered as groups of populations. The hierarchical analysis of the molecular variance, AMOVA [59], was used to test for possible genetic differentiation among classes. When less than 5% of the 10000 pseudo-replicates presented higher genetic variance than one estimated by chance, then the genetic structure was considered not significant. AMOVA also calculated the correlation measure fixation index of population differentiation (Fst). In all cases differences were considered statistically different when *P*<0.05.

## Supporting Information

Figure S1 Pattern of observed sex ratio responses to temperature in the European sea bass. Resulting number of phenotypic females as a function of the rearing temperature during the thermosensitive period (up to ∼60 days post fertilization). Data from different studies [20] with different families and expressed as mean ± S.E.M. of n ∼5 trials for each temperature. The 13–17°C range corresponds to the natural range of temperatures during sea bass spawning and larval development, and explains why at these temperatures sex ratios approach the 1∶1 Fisherian sex ratio. In contrast, 21–22°C is the commonly used temperature for larval rearing during sea bass farming. Thus, an increase of only 4°C is able to result in strongly male-biased sex ratios.
(PPT)

Figure S2 Diagram of the sea bass (sb) gonadal aromatase (*cyp19a*) promoter region analyzed in this study. Genomic DNA was extracted from the gonads of individual fish. Restriction enzyme digestion (*Bgl*II and *Dra*II) outside the region of interest was used to obtain a smaller and linearized fragment of the promoter. After bisulphite treatment, external and nested PCR was carried out to amplify a 598 bp PCR fragment. Inside this region, putative transcription factors binding sites as well as CpG dinucleotide localizations (lollipops) are shown, indicating their nucleotide position with respect to the transcription start site. Transcription and translation starting sites are symbolized with an arrow and an asterisk, respectively. Abbreviations for binding sites: Fox, forkhead transcription factor; Sox, *Sry*-related transcription factor; Are, androgen response element; SF1, steroidogenic factor-1; Ppar, peroxisome proliferation activated receptor; Cre, cAMP response element; TATA, TATA box.
(PPT)

Figure S3 Thermal protocols applied in the present study. The experimental groups (carried out in duplicate) were: Low temperature, LT, 15°C from 0–60 dpf, thereafter following the natural fluctuation; and high temperature, HT, 15°C from 0–10 dpf, then at 21°C throughout the thermosensitive period (TSP). The TSP and the sex differentiation period are indicated (with a dashed line and a line between arrows, respectively) in relation to the thermal regimens. The dashing pattern indicates that the effects of temperature are more evident shortly after fertilization and progressively wear out. The major events related to gonad formation and sex differentiation are also indicated.
(PPT)

Figure S4 Frequency distribution of average *cyp19a* promoter DNA methylation levels in relation to phenotypic sex in the European sea bass. Sex-specific differences in *cyp19a* promoter methylation in adult sea bass gonads. The dashed line indicates the methylation threshold (67%) calculated with the 95% confidence interval, which separates typical sea bass female and male *cyp19a* methylation levels.
(PPT)

Figure S5 Absolute frequency of the 58 methylation patterns observed (out of the 128 theoretically possible methylation patterns) according to sex and temperature treatment. Yellow circle, females at low temperature (FLT); red square, females at high temperature (FHT); light blue square, males at low temperature (MLT); dark blue triangle, males at high temperature (MHT).
(PPT)

Figure S6 Number of different methylation patterns observed. A, distributed according to sex and temperature treatments, and B, in relation to levels of *cyp19a* promoter methylation. Boxed numbers in panel A are the average number of observed methylation patterns in each group. Yellow circle, females at low temperature (FLT); red square, females at high temperature (FHT); light blue square, males at low temperature (MLT); dark blue triangle, males at high temperature (MHT).
(PPT)
