# Supplementary figures and images for: DNA Methylation of the Gonadal Aromatase (cyp19a) Promoter Is Involved in Temperature-Dependent Sex Ratio Shifts in the European Sea Bass
Source: PLoS Genet. 2011 Dec 29;7(12):e1002447. doi: 10.1371/journal.pgen.1002447 (PMC3248465; doi:10.1371/journal.pgen.1002447)

## Slide 1
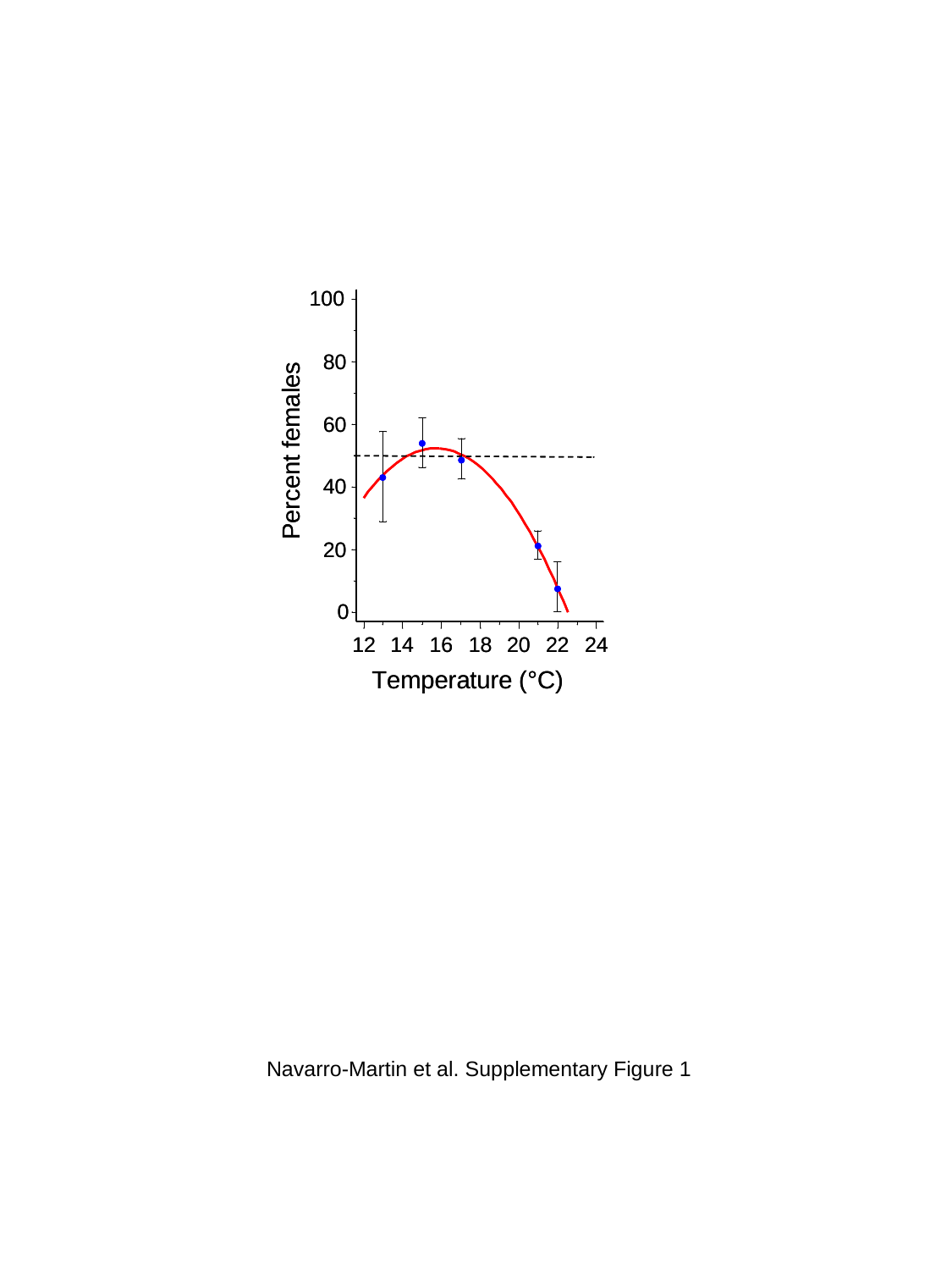

Navarro-Martin et al. Supplementary Figure 1

Supplement: Figure S1 — Pattern of observed sex ratio responses to temperature in the European sea bass. Resulting number of phenotypic females as a function of the rearing temperature during the thermosensitive period (up to ∼60 days post fertilization). Data from different studies [20] with different families and expressed as mean ± S.E.M. of n ∼5 trials for each temperature. The 13–17°C range corresponds to the natural range of temperatures during sea bass spawning and larval development, and explains why at these temperatures sex ratios approach the 1∶1 Fisherian sex ratio. In contrast, 21–22°C is the commonly used temperature for larval rearing during sea bass farming. Thus, an increase of only 4°C is able to result in strongly male-biased sex ratios. (PPT) [file pgen.1002447.s001.ppt]

## Slide 1
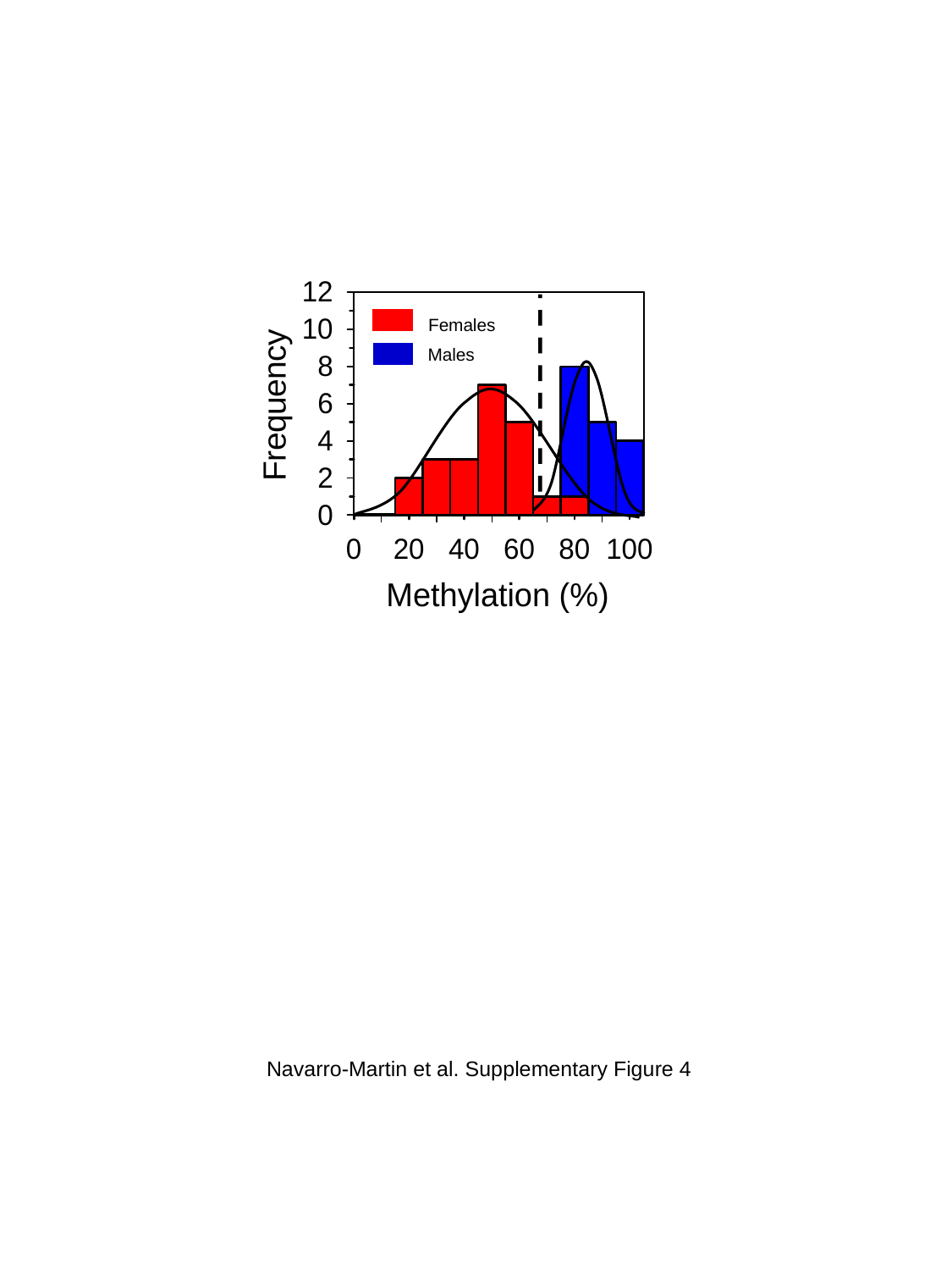

Females
Males
Navarro-Martin et al. Supplementary Figure 4

Supplement: Figure S4 — Frequency distribution of average cyp19a promoter DNA methylation levels in relation to phenotypic sex in the European sea bass. Sex-specific differences in cyp19a promoter methylation in adult sea bass gonads. The dashed line indicates the methylation threshold (67%) calculated with the 95% confidence interval, which separates typical sea bass female and male cyp19a methylation levels. (PPT) [file pgen.1002447.s004.ppt]

## Slide 1
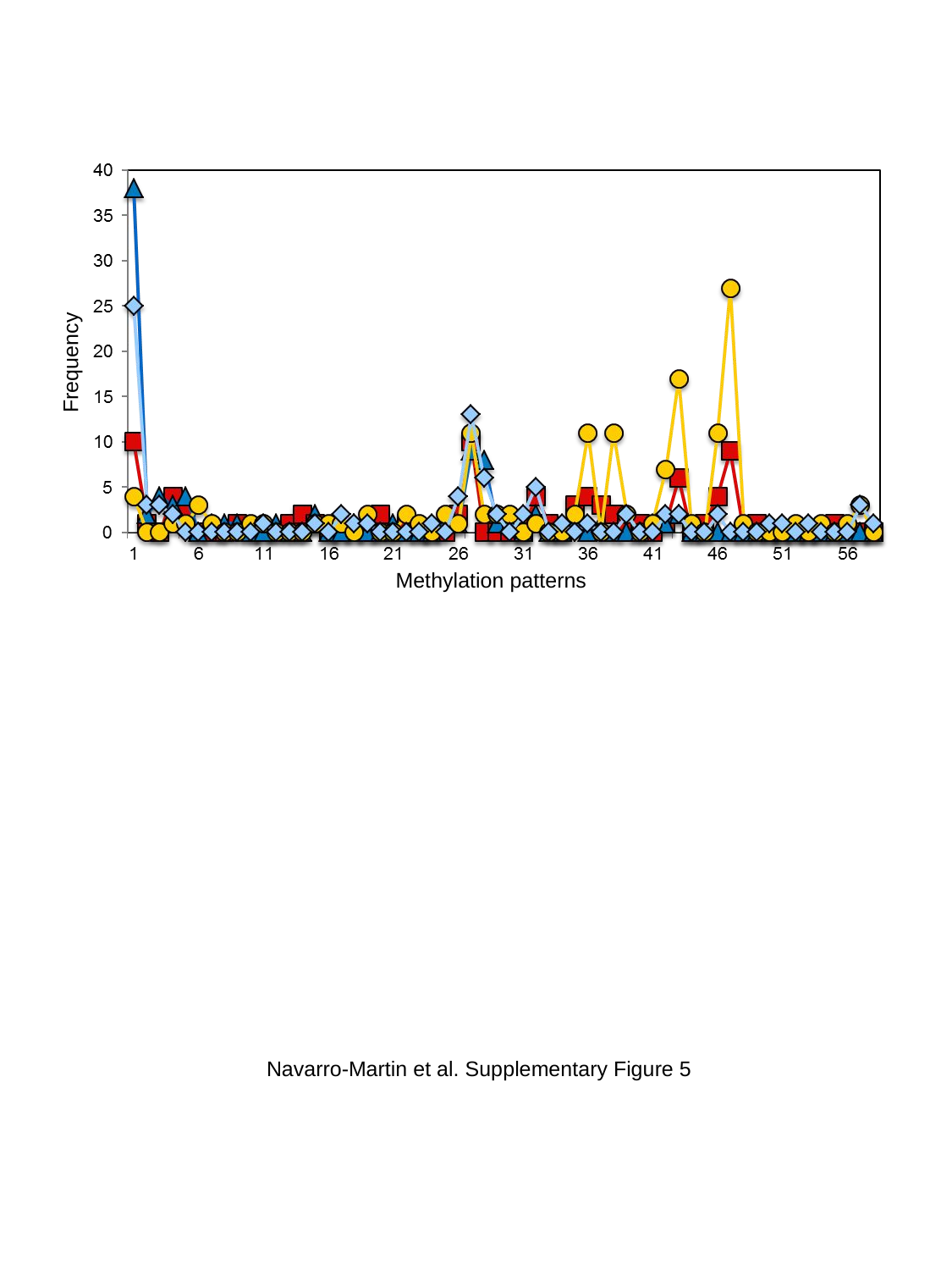

Frequency
Methylation patterns
Navarro-Martin et al. Supplementary Figure 5

Supplement: Figure S5 — Absolute frequency of the 58 methylation patterns observed (out of the 128 theoretically possible methylation patterns) according to sex and temperature treatment. Yellow circle, females at low temperature (FLT); red square, females at high temperature (FHT); light blue square, males at low temperature (MLT); dark blue triangle, males at high temperature (MHT). (PPT) [file pgen.1002447.s005.ppt]
