# Supplementary material for: DNA Methylation of the Gonadal Aromatase (cyp19a) Promoter Is Involved in Temperature-Dependent Sex Ratio Shifts in the European Sea Bass
Source: PLoS Genet. 2011 Dec 29;7(12):e1002447. doi: 10.1371/journal.pgen.1002447 (PMC3248465; doi:10.1371/journal.pgen.1002447)
Supplement: Figure S2 — Diagram of the sea bass (sb) gonadal aromatase (cyp19a) promoter region analyzed in this study. Genomic DNA was extracted from the gonads of individual fish. Restriction enzyme digestion (BglII and DraII) outside the region of interest was used to obtain a smaller and linearized fragment of the promoter. After bisulphite treatment, external and nested PCR was carried out to amplify a 598 bp PCR fragment. Inside this region, putative transcription factors binding sites as well as CpG dinucleotide localizations (lollipops) are shown, indicating their nucleotide position with respect to the transcription start site. Transcription and translation starting sites are symbolized with an arrow and an asterisk, respectively. Abbreviations for binding sites: Fox, forkhead transcription factor; Sox, Sry-related transcription factor; Are, androgen response element; SF1, steroidogenic factor-1; Ppar, peroxisome proliferation activated receptor; Cre, cAMP response element; TATA, TATA box. (PPT) [file pgen.1002447.s002.ppt]

## Slide 1
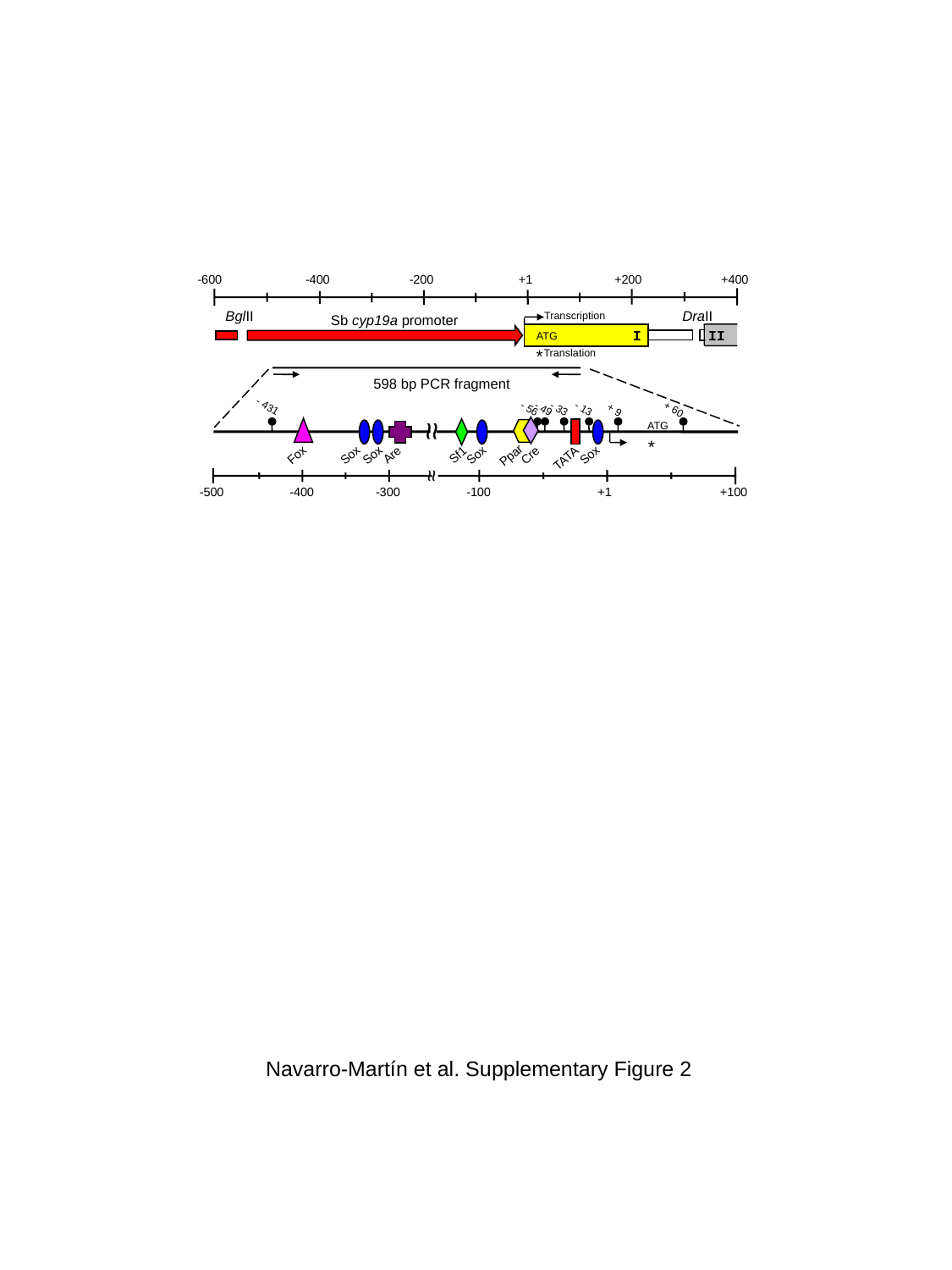

-600
-400
-200
+1
+200
+400
BglII
DraII
Transcription
Sb cyp19a promoter
I
II
ATG
*
Translation
598 bp PCR fragment
- 431
- 33
- 13
- 49
+ 9
- 56
+ 60
≈
ATG
*
Cre
Sox
Sox
Are
Sf1
Sox
Sox
Fox
Ppar
TATA
≈
-500
-400
-300
-100
+1
+100
Navarro-Martín et al. Supplementary Figure 2
