# Supplementary material for: DNA Methylation of the Gonadal Aromatase (cyp19a) Promoter Is Involved in Temperature-Dependent Sex Ratio Shifts in the European Sea Bass
Source: PLoS Genet. 2011 Dec 29;7(12):e1002447. doi: 10.1371/journal.pgen.1002447 (PMC3248465; doi:10.1371/journal.pgen.1002447)
Supplement: Figure S3 — Thermal protocols applied in the present study. The experimental groups (carried out in duplicate) were: Low temperature, LT, 15°C from 0–60 dpf, thereafter following the natural fluctuation; and high temperature, HT, 15°C from 0–10 dpf, then at 21°C throughout the thermosensitive period (TSP). The TSP and the sex differentiation period are indicated (with a dashed line and a line between arrows, respectively) in relation to the thermal regimens. The dashing pattern indicates that the effects of temperature are more evident shortly after fertilization and progressively wear out. The major events related to gonad formation and sex differentiation are also indicated. (PPT) [file pgen.1002447.s003.ppt]

## Slide 1
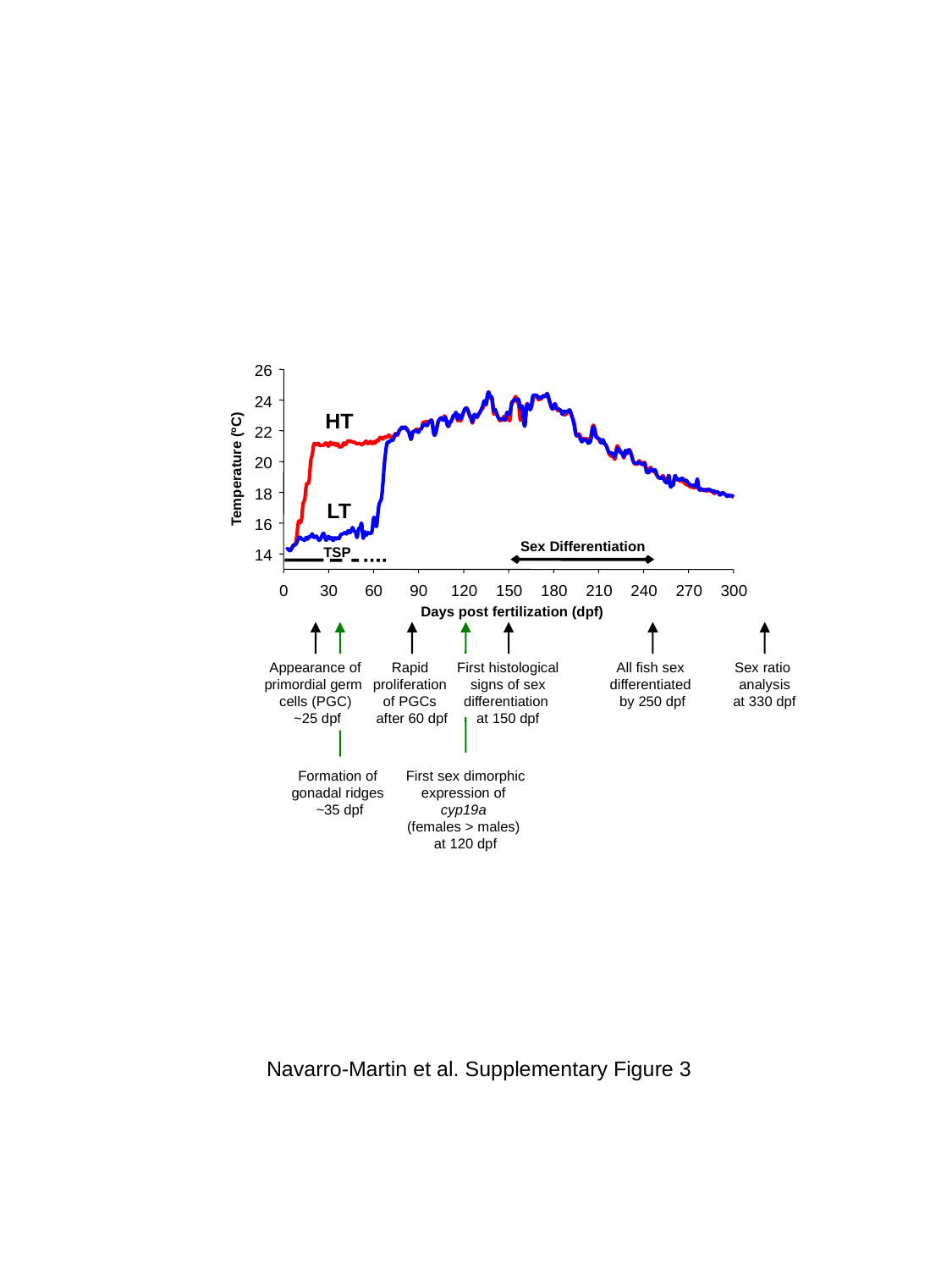

26
24
HT
22
20
Temperature (ºC)
18
LT
16
Sex Differentiation
TSP
14
0
30
60
90
120
150
180
210
240
270
300
Days post fertilization (dpf)
Appearance of
primordial germ
cells (PGC)
 ~25 dpf
Rapid
proliferation
of PGCs
after 60 dpf
First histological
signs of sex
differentiation
at 150 dpf
All fish sex
differentiated
by 250 dpf
Sex ratio
analysis
at 330 dpf
Formation of
gonadal ridges
~35 dpf
First sex dimorphic
expression of
cyp19a
(females > males)
at 120 dpf
Navarro-Martin et al. Supplementary Figure 3
