# Supplementary material for: DNA Methylation of the Gonadal Aromatase (cyp19a) Promoter Is Involved in Temperature-Dependent Sex Ratio Shifts in the European Sea Bass
Source: PLoS Genet. 2011 Dec 29;7(12):e1002447. doi: 10.1371/journal.pgen.1002447 (PMC3248465; doi:10.1371/journal.pgen.1002447)
Supplement: Figure S6 — Number of different methylation patterns observed. A, distributed according to sex and temperature treatments, and B, in relation to levels of cyp19a promoter methylation. Boxed numbers in panel A are the average number of observed methylation patterns in each group. Yellow circle, females at low temperature (FLT); red square, females at high temperature (FHT); light blue square, males at low temperature (MLT); dark blue triangle, males at high temperature (MHT). (PPT) [file pgen.1002447.s006.ppt]

## Slide 1
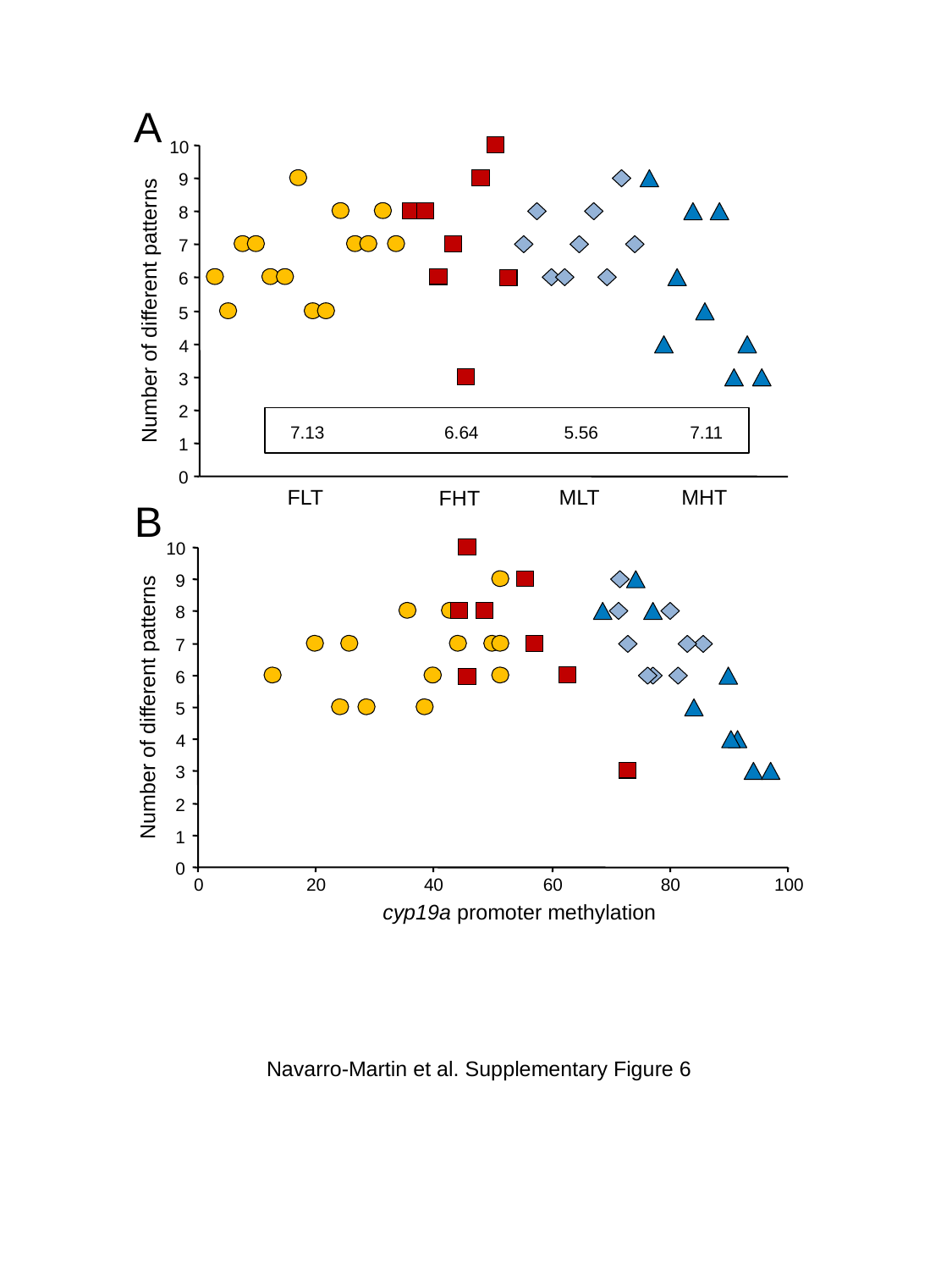

A
10
9
8
7
6
Number of different patterns
5
4
3
2
7.13
6.64
5.56
7.11
1
0
MHT
FLT
MLT
FHT
B
10
9
8
7
6
Number of different patterns
5
4
3
2
1
0
0
20
40
60
80
100
cyp19a promoter methylation
Navarro-Martin et al. Supplementary Figure 6
